# *SLG2* specifically regulates grain width through *WOX11* ‐mediated cell expansion control in rice

**DOI:** 10.1111/pbi.14102

**Published:** 2023-06-21

**Authors:** Dunpin Xiong, Ruci Wang, Yueming Wang, Yi Li, Ge Sun, Shanguo Yao

**Affiliations:** ^1^ State Key Laboratory of Plant Genomics, Institute of Genetics and Developmental Biology, The Innovative Academy of Seed Design Chinese Academy of Sciences Beijing China; ^2^ University of Chinese Academy of Sciences Beijing China

**Keywords:** *SLG2*, *WOX11*, *GW8*, grain width, cell expansion, rice

## Abstract

Grain size is specified by three dimensions of length, width and thickness, and slender grain is a desirable quality trait in rice. Up to now, many grain size regulators have been identified. However, most of these molecules show influence on multi‐dimensions of grain development, and only a few of them function specifically in grain width, a key factor determining grain yield and appearance quality. In this study, we identify the *SLG2* (*SLENDER GUY2*) gene that specifically regulates grain width by affecting cell expansion in the spikelet hulls. *SLG2* encodes a WD40 domain containing protein, and our biochemical analyses show that SLG2 acts as a transcription activator of its interacting WOX family protein WOX11. We demonstrate that the SLG2‐associated WOX11 binds directly to the promoter of *OsEXPB7*, one of the downstream cell expansion genes. We show that knockout of *WOX11* results in plants with a slender grain phenotype similar to the *slg2* mutant. We also present that finer grains with different widths could be produced by combining *SLG2* with the grain width regulator *GW8*. Collectively, we uncover the crucial role of *SLG2* in grain width control, and provide a promising route to design rice plants with better grain shape and quality.

## Introduction

Rice is one of the most important cereal crops and the stable food of nearly half of the world's people. With the increase of the world population, the deterioration of the environment and the reduction of the arable land, increasing grain yield remains the major challenge for most rice growing areas. Rice grain yield is composed of three major factors: panicle number, grain number per panicle, and grain weight. The grain weight is determined by grain size and grain filling. Because grain size is a key determinant of yield and appearance quality, it is one of the major targets of artificial selection during domestication, and is an important trait for rational design of high‐yield and high‐quality rice varieties.

Grain size is specified by three dimensions of length, width and thickness. Based on the analysis of mutant materials and natural populations, nearly 200 genes have been cloned for their functions in grain size regulation (Jiang *et al*., 2022). Functional study of these genes revealed a variety of regulatory routes involved in rice grain size control. For example, *SMALL GRAIN 1* (*OsSMG1*)/(*OsMKK4*), *OsMAPK6* and *OsMKKK10* control grain size through the mitogen‐activated protein kinase signalling pathway (Duan *et al*., 2014; Liu *et al*., 2015a,b; Xu *et al*., 2018), and *GS3*, *RGB1*, *DEP1* and *RGG2* are important grain size regulators in the G protein signalling pathway (Fan *et al*., 2006; Miao *et al*., 2019; Utsunomiya *et al*., 2011; Xu *et al*., 2016). In addition, *WIDE AND THICK GRAIN 1* (*WTG1*)/(*OsOTUB1*), *GW2* and *OsUBP15* affect grain size by the ubiquitin‐proteasome pathway (Choi *et al*., 2018; Huang *et al*., 2017; Shi *et al*., 2019), while *GS2*, *GW8*, *GLW7* and *OsPIL15* determine grain size through transcriptional factors‐mediated pathway (Hu *et al*., 2015; Si *et al*., 2016; Sun *et al*., 2020; Wang *et al*., 2015). Besides, *GS5*, *BG1* and *GW5* are vital grain size controllers involved in the phytohormone signalling pathway (Li *et al*., 2011; Liu *et al*., 2015a,b). Moreover, regulators such as *OsmiR156*, *OsmiR396*, *OsmiR408* and *OsAGO17* appear to define grain size in the epigenetic pathway (Duan *et al*., 2015; Jiao *et al*., 2010; Zhang *et al*., 2017; Zhong *et al*., 2020a,b).

Because slender grains are preferred by the majority of consumers (Wang *et al*., 2012), isolation of regulators that only affect one of the three dimensions of grain size, especially grain width, is particularly important for rice grain yield and appearance quality (Jiang *et al*., 2022). So far, mutational analysis in rice has identified many genes controlling grain size (Jiang *et al*., 2022). However, except for *GS6* (Sun *et al*., 2013) and *GW5* (Tian *et al*., 2019), most of the qualitative loci isolated from grain size mutants are difficult to meet the actual production demands due to adverse effects on other agronomic traits (Chen *et al*., 2021). By studying germplasms with different grain shapes, about 22 quantitative trait loci (QTLs) have been identified to regulate grain size in rice (Jiang *et al*., 2022). However, a careful survey of the phenotypes of the loss of function mutants, but not near isogenic lines or knockdown/overexpression transgenic lines, revealed that most of these genes show influence on the development of grains in multi‐dimensions, and only four genes *GS5* (Li *et al*., 2011), *GW5* (Tian *et al*., 2019), *TGW2* (Ruan *et al*., 2020) and *TGW12a* (Du *et al*., 2021) display a regulatory role confined to grain width. However, the application value of these four genes in regulating grain width remains to be evaluated, as they all seem to affect the grain filling process. Up to now, *GW8*, a squamosa promoter binding protein coding gene that has been artificially selected in the rice breeding program, should be the only regulatory factor that can improve the appearance quality of rice without adversely affecting other grain traits (Wang *et al*., 2012). The ultimate goal of studying grain size genes is undoubtedly for breeding, and pyramiding of multiple genes is usually necessary to obtain plants with ideal grain size and appearance quality (Jiang *et al*., 2022). However, one can imagine that if the identified regulators affect multiple dimensions of grain development, it would be difficult to achieve a balance among grain size, grain filling and grain appearance during gene pyramiding. In other words, we need to excavate more regulators that only affect one of the three dimensions of grain size, especially grain width.

The WD40 repeats are one of the most abundant domains in the eukaryotic genome, and members of the WD40 family act as scaffolds for protein–protein interactions (Jain and Pandey, 2018; Stirnimann *et al*., 2010). In plants, the WD40 proteins take part in diverse processes such as immune response, cell wall formation, histone modification, proteasomal degradation, and microtubule organization (Jain and Pandey, 2018). The rice genome is predicted to contain 231 genes coding for WD40 protein (Yang *et al*., 2021). Genetic analysis of these OsWD40s has revealed their versatile functions in multiple developmental processes, such as grain number (Chen *et al*., 2022), anthocyanin biosynthesis (Yang *et al*., 2021), pollen tube germination and elongation (Kim *et al*., 2021), flowering and panicle branching (Jiang *et al*., 2018), and fertility (Qin *et al*., 2016). And, mutant analysis also has uncovered the role of several *OsWD40s* in grain development, such as *OsFIE1* (Cheng *et al*., 2020), *OsFIE2* (Nallamilli *et al*., 2013), *OsLIS‐L1* (Gao *et al*., 2012), *RGB1* (Utsunomiya *et al*., 2011; Zhang et al., 2021a,b,c), and *DRW1* (Zhang et al., 2021a,b,c). However, due to the numerous roles of WD40 proteins in cellular processes, dysfunction of these genes often leads to malformed development of not only grain size but also many other organs. Recently, Dhatt *et al*. (2021) reported the contribution of the allelic variation in *OsFIE1* to grain width under high night temperature stress. As far as we know, this should be the only report linking WD40 protein with rice grain width so far. Because the functions of most OsWD40 members are still unclear, much effort should be made to reveal their roles in grain size regulation.

In this study, we report the identification of *slg2*, a rice mutant with specifically reduced grain width caused by decreased cell expansion. We show that SLG2 regulates grain width by recruiting and activating the WOX family transcription factor WOX11 to directly promote the expression of downstream cell expansion genes. We also demonstrate that the combination of *SLG2* with another grain width regulator, *GW8*, could produce finer grains with different widths. Our results suggest that the grain width‐specific regulator *SLG2* has potential value in designing rice plants with better grain shape and quality.

## Results

### Rice mutant *slg2* shows a specific decrease in grain width

**Figure 1:**
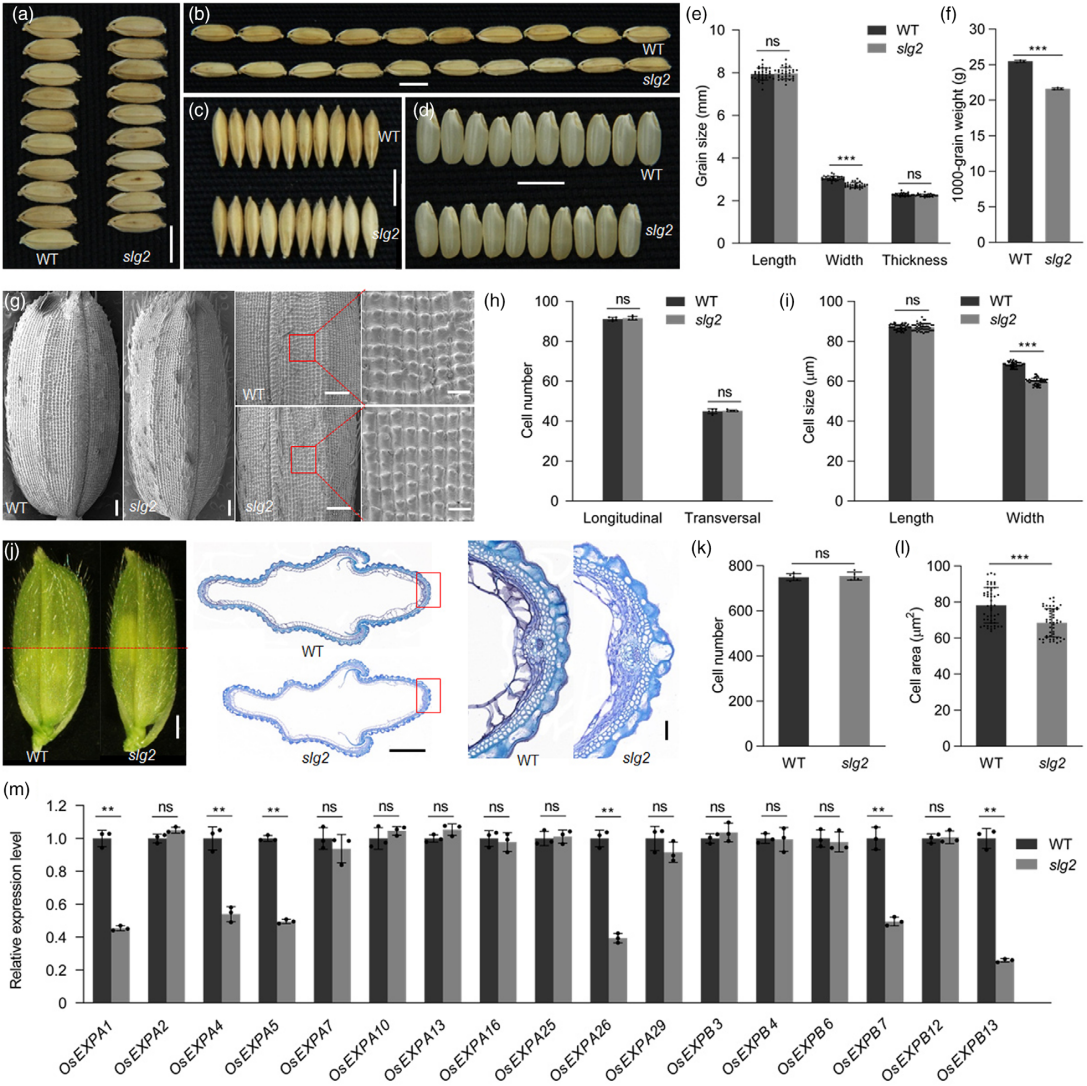
Characterization of the *slg2* mutant. (a–d) Comparison of grain width (a), grain length (b), grain thickness (c) and grain quality (d) between *slg2* and WT. Bars = 5 mm. (e, f) Statistical analysis of grain size (e) and 1000‐grain weight (f) between *slg2* and WT. Data are means ± SD (*n* = 30 for e and 3 for f). ***: *P* < 0.001, ns: no significant difference (Student's *t* test). (g–i) SEM observation of outer glume surfaces (g) and statistical analysis of cell number (h) and cell size (i) in *slg2* and WT. For (g), bars = 500 μm for the left images, and 50 μm for the right images. Data are means ± SD (*n* = 5 for h and 50 for i). ***: *P* < 0.001, ns: no significant difference (Student's *t* test). (j–l) Cross‐sections of the spikelet hulls (j) and statistical analysis of cell number (k) and cell area (l) in *slg2* and WT. The spikelet hulls are sampled before anthesis. In (j), the dotted line indicates the sites of the cross‐sections shown in middle, and the boxes indicate the sites of the magnified images shown in left. Bars = 1 mm (left), 500 μm (middle) and 50 μm (right). Data are means ± SD (*n* = 5 for k and 50 for l). ***: *P* < 0.001, ns: no significant difference (Student's *t* test). (m) Expression analysis of cell expansion genes in *slg2* and WT. RNA isolated from young panicles of 2–3 mm in length is used for RT‐qPCR. *OsActin* is used as the internal control. The transcript levels are normalized against WT, which is set to 1. Data are means ± SD (*n* = 3). **: *P* < 0.01, ns: no significant difference (Student's *t* test).

By screening the NaN_3_‐mutagenized M_2_ library in the background of ZY66 (*japonica*), we identified a mutant with obviously decreased grain width (Figure 1a,d), and the mutant was then designated *slg2* (*slender guy 2*). Compared with the wild type (WT), the grain width of *slg2* was reduced by about 10.5% (Figure 1a,d,e). In contrast, the other two dimensions of grain size, i.e., grain length and grain thickness, remained similar between *slg2* and WT (Figure 1b,c,e). In addition, we paid special attention to grain chalkiness, which is an important trait closely related to grain quality, especially appearance quality. We found that there was no difference in the transparency of brown rice endosperm between *slg2* and WT (Figure 1d; Figure S1a). We further observed the starch granules by scanning electron microscopy (SEM), and found that the starch packaging in the endosperm of *slg2* was similar to that of WT (Figure S1b). We also investigated the grain filling by monitoring caryopsis development of the mutant during the whole grain maturation stage (Figure S2a). No difference was observed for ovary extension in the longitudinal direction between *slg2* and WT. However, from 5 days after fertilization (DAF), the caryopsis growth of the mutant lagged behind WT in the transversal direction (Figure S2a), and the grain weight of *slg2* was obviously lower than that of WT (Figure S2b,c). Nonetheless, the trend of grain weight increase during caryopsis maturation was similar between the mutant and WT (Figure S2b,c), and the grain filling rate of *slg2* was identical to that of WT (Figure S2d). Taken together, these results suggest that *SLG2* is a grain size regulator that acts specifically on grain width, but not on grain length, grain thichness or grain filling.

Grain size is determined by the spikelet hull composed of palea and lemma, and the development of spikelet hull is coordinately regulated by cell proliferation and cell expansion (Li *et al*., 2018). To understand the cellular process of *SLG2* regulating grain size, we performed SEM observation of epidermal cells in the lemma of *slg2* and WT (Figure 1g). We found that cell number in the central part of the lemma of *slg2* was comparable to that of WT both in the longitudinal and transversal directions (Figure 1h). The length of the epidermal cells was similar between *slg2* and WT. In contrast, the width of these cells in *slg2* was about 12.4% less than that in WT (Figure 1g,i). The decrease of cell width in *slg2* coincided well with the reduction of grain width in the mutant (Figure 1e,i). We further carried out cytological observation using cross‐sections of the central part of spikelet hulls before anthesis (Figure 1j). In agreement with the result of SEM observation, the number of cells in the outer parenchyma cell layer of *slg2* remained unchanged (Figure 1k), but the size of these cells was reduced by about 12.2% compared with WT (Figure 1l).

To gain insight into the molecular basis of *SLG2*‐mediated grain size control, we performed expression analysis of genes related to cell division and cell expansion. We found no significant difference in the transcripts levels of genes known to function in G1‐to‐S transition (*H1*, *E2F2*, *CAK1*, *CDKA1* and *MCM3*) and G2‐to‐M transition (*CYCA2.1*, *CYCB2.1*, *CYCB2.2* and *CDKB*) between *slg2* and WT (Figure S3a). Of the 17 cell expansion genes that appear to express in young panicles (https://ricexpro.dna.affrc.go.jp/GGEP/), six genes (*OsEXPA1*, *OsEXPA4*, *OsEXPA5*, *OsEXPA26*, *OsEXPB7* and *OsEXPB13*) showed suppressed transcription in *slg2* relative to WT (Figure 1m). We also explored the possible regulatory relationship between *SLG2* and previously identified grain width‐related genes by detecting the expression level of these genes, including *GW2* (Song *et al*., 2007), *GS5* (Li *et al*., 2011), *GS6* (Sun *et al*., 2013), *GW8* (Wang *et al*., 2012), *GW5* (Liu *et al*., 2017), *GW5L* (Tian *et al*., 2019), *TGW2* (Ruan *et al*., 2020), *GW6* (Shi *et al*., 2020) and *TGW12a* (Du *et al*., 2021). We did not find significant differences in the transcripts abundance of these genes between *slg2* and WT (Figure S3b). Taken together, these results suggest that *SLG2* controls grain width by affecting cell expansion in the spikelet hull in a way independent of the currently identified grain width genes.

A survey of other agronomic traits showed that the overall development of *slg2* was basically normal (Figure S4a). The heading date, tillering ability and seed setting rate of *slg2* were identical with those of WT (Figure S4c,d,f), while plant height of the mutant was about 3.5 cm lower than that of WT (Figure S4b). The difference of agronomic traits between *slg2* and WT, in addition to grain width, was mainly the number of grains per panicle (85.3 vs. 96.6; Figure S4e). As a result, the grain yield of the mutant decreased by about 15% compared with that of WT (Figure S4g).

### *SLG2* encodes a WD40 domain containing protein

**Figure 2:**
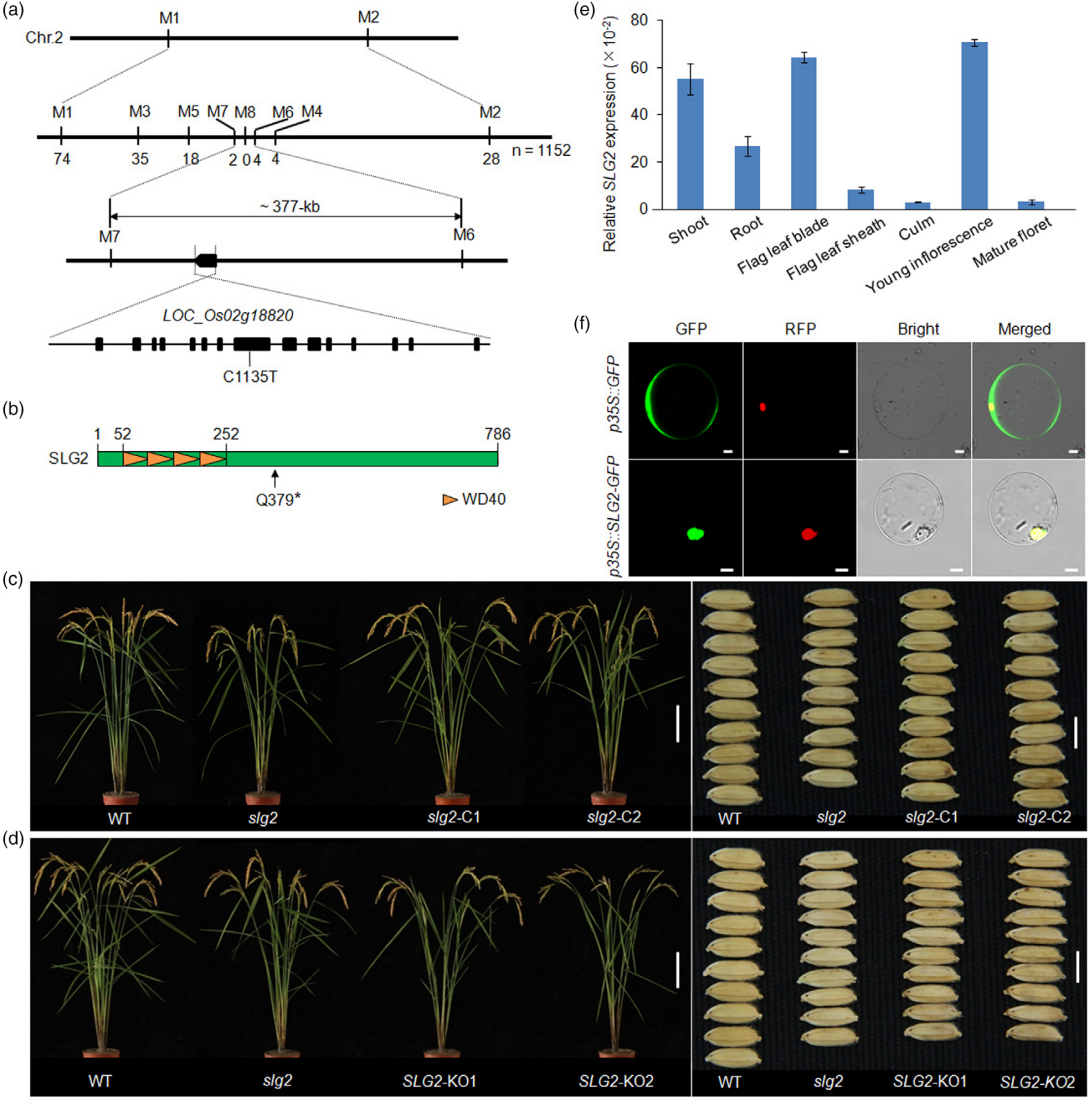
Map‐based Cloning of *SLG2*. (a, b) Identification of the *SLG2* candidate gene. Indicating one C‐T point mutation occurred in *LOC_Os02g18820* in the *slg2* mutant, which generates a premature stop codon. (c, d) Phenotypes of whole plant and grain size of WT, *slg2*, complementation lines (*slg2*‐C) and knockout lines (*SLG2*‐KO). Bars = 15 cm for whole plant and 5 mm for grain size. (e) Expression analysis of *SLG2* in various rice tissues. For RT‐qPCR, RNA is isolated from shoots and roots of 7‐day‐old seedlings, and flag leaf blade, flag leaf sheath, culm, young panicle (2–3 mm in length) and mature floret before anthesis. *OsActin* is used as the internal control. Data are means ± SD (*n* = 3). (f) Subcellular localization of SLG2. Bars = 10 μm.

To understand the molecular mechanism of *SLG2*‐mediated grain width control, we performed map‐based cloning to isolate the causal gene. Crossing of *slg2* with WT yielded an F_2_ population, in which the segregation ratio of WT and slender grain phenotype was 3:1 (398:128; χ^2^ = 0.12; 0.5 > *P* > 0.1). This result suggests that the *slg2* mutant phenotype is caused by the recessive mutation of a single locus. By using 20 bulked WT and *slg2* mutant plants from the F_2_ population between *slg2* and KY131 (*japonica*), the causal gene was initially mapped to the short arm of chromosome 2 between markers M1 and M2 (Figure 2a). And, the candidate was further narrowed down to a 377‐kb region between markers M6 and M7 using 1152 F_2_ individuals with a mutant phenotype (Figure 2a). After sequencing the fine mapped region, we found that a point mutation of C to T occurred in *slg2* in the eighth exon of the annotated gene *LOC_Os02g18820* (Figure 2a; http://rice.plantbiology.msu.edu/), leading to the formation of a premature stop codon in the mutant (Figure 2b).

To verify that the C‐T substitution was responsible for the *slg2* mutant phenotype, a 17.8‐kb genomic sequence of *LOC_Os02g18820*, including the entire coding region, 2392‐bp upstream of ATG and 1567‐bp downstream of TGA, was amplified from WT and introduced into the *slg2* mutant. We found that the overall phenotypes of all the 28 T_0_ positive transgenic plants, including grain width and cell width of the spikelet hull, resembled that of WT (Figure 2c; Figure S5). In addition, by selecting a specific target site in the third exon (Figure S6a), we tried to knock out the *LOC_Os02g18820* locus in the ZY66 background using the CRISPR/Cas9 genome editing tool. We obtained a series of transgenic lines with multiple types of insertions or deletions in the coding region, and selected two representative lines with 1‐bp (*SLG2*‐KO1) and 2‐bp (*SLG2*‐KO2) insertions for phenotypic analysis (Figure S6a). We noticed that similar to the *slg2* mutant, both *SLG2*‐KO lines presented slender grain phenotype (Figure 2d; Figure S6c). Moreover, we found that cell width of the spikelet hull of the *SLG2*‐KO plants was reduced (Figure S6b,d), and transcription of the cell expansion genes differentially expressed in *slg2* were also suppressed in the *SLG2*‐KO lines (Figure S6e). We further created *SLG2*‐overexpressing transgenic lines in the background of *slg2* (*SLG2*‐OE^*slg2*^). Consistent with the results of the complementation test, overexpression of *SLG2* fully rescued the overall phenotypes of the mutant (Figure S7a,b), including the reduced grain width (Figure S7c,d). These observations clearly demonstrate that the *slg2* phenotypes are caused by the point mutation in *LOC_Os02g18820*.

The transcripts of *SLG2* were detected in all the tissues tested, including shoot, root, flag leaf blade, flag leaf sheath, culm, young panicle and mature floret, with the highest expression in the young panicle (Figure 2e). SLG2 is localized in the nucleus (Figure 2f), and is predicted to be a WD40 domain containing protein with four WD40 repeats in its N terminus (Figure 2b; https://www.ncbi.nlm.nih.gov/). In rice, there are 231 *OsWD40s* in the genome (Yang *et al*., 2021), and *LOC_Os09g12550* is the only paralog of *SLG2* (http://plants.ensembl.org/index.html). Because these two genes shared an identity of about 65% at the protein level (Figure S8), and *LOC_Os09g12550* displayed a tissue‐specific expression pattern similar to *SLG2* (Figure S9a), the gene was then designated *HSLG2* (*Homologue of SLG2*). To understand whether *HSLG2* also plays a role in grain size control, we knocked out this gene in the ZY66 background by specifically editing the target site in the second exon (Figure S9b). Analysis of three independent lines with differential mutations in the coding region revealed that the *HSLG2*‐KO plants had no any alterations in overall agronomic traits including grain size (Figure S9b–f). Moreover, we created the *slg2,hslg2* double mutants by crossing *HSLG2*‐KO1 plants with *slg2*. No obvious difference was observed between *slg2,hslg2* and *slg2* in overall agronomic traits including grain width (Figure S9c–f), and the transcripts of *SLG2* remained unchanged between *HSLG2*‐KO and WT (Figure S9g). These observations suggest that *SLG2* and *HSLG2* have divergent functions in rice plant development.

### SLG2 interacts with WOX11, a positive grain width regulator

**Figure 3:**
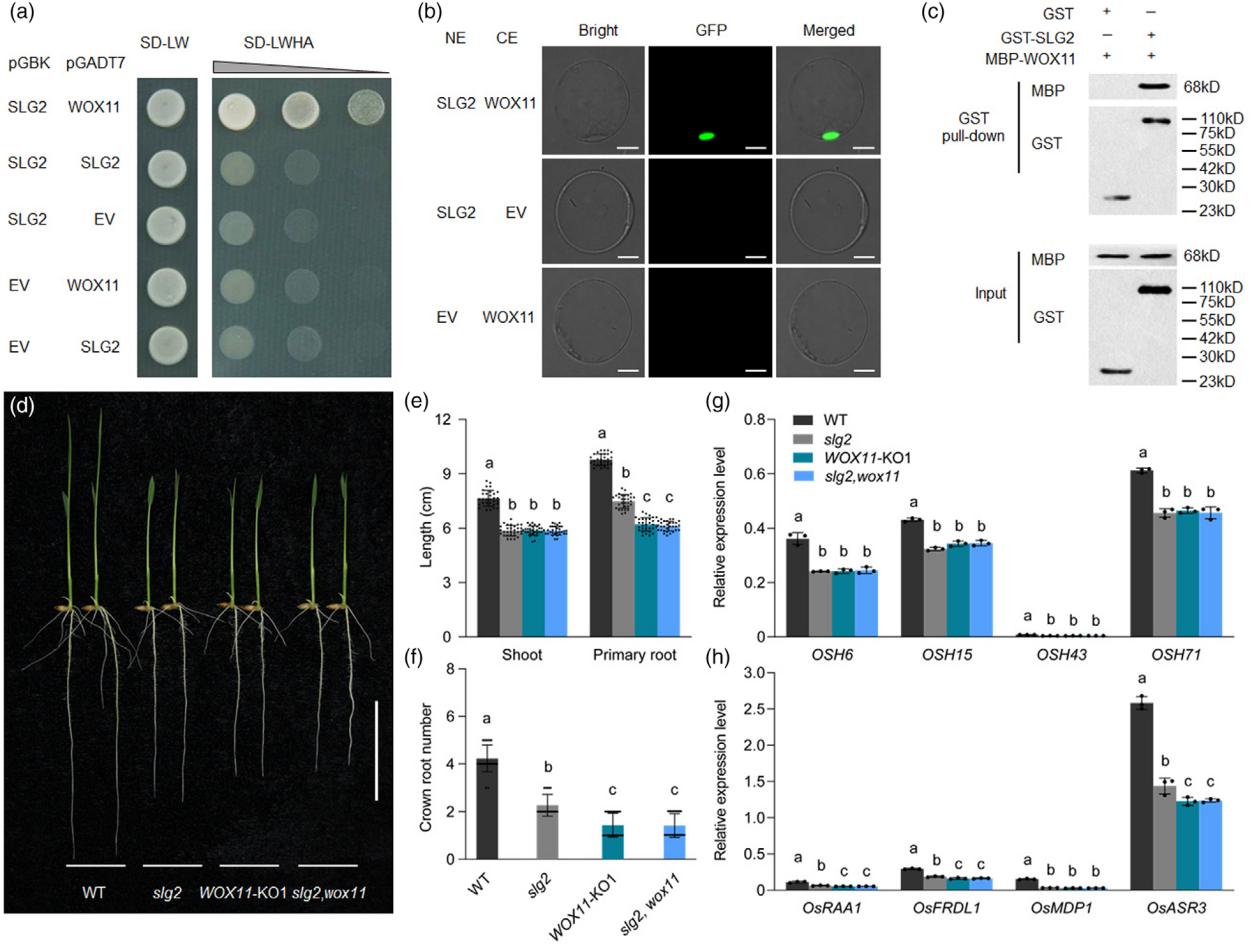
SLG2 and WOX11 interact and regulate rice seedling development. (a–c) Interaction assay between SLG2 and WOX11. Bars = 10 μm (b). (d) Morphological comparison of 7‐day‐old seedlings of WT, *slg2*, *WOX11* knockout line (*WOX11*‐KO) and *slg2,wox11* double mutant plants. Bar = 4 cm. (e, f) Statistical analysis of root length (e) and crown root number (f) of WT, *slg2*, *WOX11*‐KO, and *slg2,wox11*. Statistical analysis is performed in seedlings shown in (d). Bars followed by different letters represent significant difference at 5%. (g, h) Expression analysis of genes involved in shoot (g) and root (h) development. RNA isolated from the shoots and roots of the seedlings shown in (a) is used for RT‐qPCR. *OsActin* is used as the internal control. Data are means ± SD (*n* = 3). Bars followed by different letters represent significant difference at 5%.

In most cases, WD40 repeat containing proteins achieve their regulatory functions by associating with other transcription factors (Jain and Pandey, 2018). In order to uncover the mechanism of *SLG2*‐mediated grain width control, we used a cDNA library prepared from young panicles (2–3 mm in length) to screen the interacting proteins of SLG2 by the yeast two‐hybrid system. Among the sequenced positive colonies, we were highly interested in the potential partner WOX11 (Figure 3a), a transcription factor appeared to play important roles in multiple rice developmental processes, including crown root, shoot, plant height, and panicle size (Cheng *et al*., 2018; Jiang *et al*., 2017; Zhou *et al*., 2017). In fact, except for the reduced plant height and panicle size (Figure S4), we also observed that at the seedling stage, the growth of shoot and root of *slg2* was obviously slower than that of WT (Figure 3d–f). Moreover, we found that in the *slg2* mutant, transcription of genes related to the development of shoots (such as *OSH6*, *OSH15*, *OSH43* and *OSH71*) and roots (such as *OsRAA1*, *OsFRDL1*, *OsMDP1* and *OsASR3*) was markedly suppressed (Figure 3g,h). All these genes have been reported to be positively regulated by *WOX11* (Cheng *et al*., 2018; Jiang *et al*., 2017). Although it remained unclear whether *WOX11* plays a role in grain size regulation, the highly similar functions of *SLG2* and *WOX11* in multiple developmental processes strongly suggest that SLG2 may control grain width by associating with the transcription factor WOX11. To verify this speculation, we first confirmed the interaction between SLG2 and WOX11 by a bimolecular fluorescence complementation (BiFC) assay in the rice protoplast (Figure 3b). In addition, *in vitro* pull‐down assay indicated that only GST‐SLG2, but not free GST, was able to pull down MBP‐WOX11 (Figure 3c). We thus concluded that SLG2 physically interacts with WOX11, both *in vivo* and *in vitro*.

**Figure 4:**
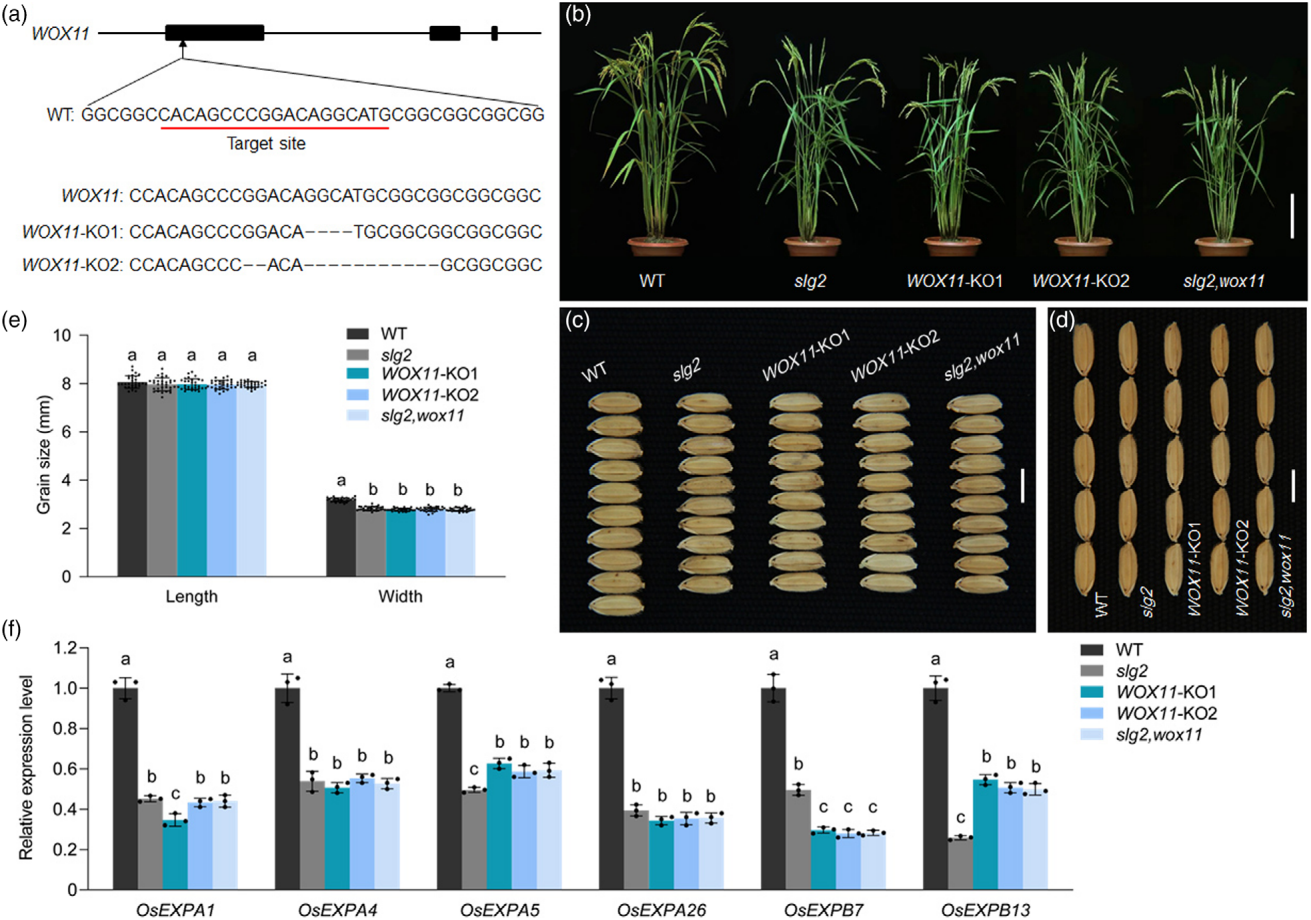
The wox*11* mutant displays slender grain phenotype similar to *slg2*. (a) Creation of *WOX11* knockout transgenic line by the CRISPR‐Cas9 genome editing system. The target site is red‐underlined. The representative transgenic lines (abbreviated as *WOX11*‐KO1 and *WOX11*‐KO2, respectively) are generated in ZY66 genetic background. The dashes indicate deleted nucleotides. (b) Plant morphology of WT, *slg2*, *WOX11*‐KO and *slg2,wox11* at the maturation stage. Bar = 15 cm. (c, d) Comparison of grain width (c) and grain length (d) among WT, *slg2*, *WOX11*‐KO and *slg2,wox11*. Bars = 5 mm. (e) Statistical analysis of grain length and grain width in WT, *slg2*, *WOX11*‐KO and *slg2,wox11*. Data are means ± SD (*n* = 30). Bars followed by different letters represent significant difference at 5%. (f) Expression analysis of cell expansion genes in WT, *slg2*, *WOX11*‐KO and *slg2,wox11*. RNA isolated from young panicles of 2–3 mm in length is used for RT‐qPCR. *OsActin* is used as the internal control. The transcript levels are normalized against WT, which is set to 1. Data are means ± SD (*n* = 3). Bars followed by different letters represent significant difference at 5%.

To reveal the role of *WOX11* in grain size regulation, we tried to knock out the locus in the ZY66 background by specifically editing a target site in the first exon (Figure 4a), and obtained dozens of transgenic plants with multiple types of mutations in the coding region. Among these transgenic plants, we selected two homozygous lines with 4‐ and 13‐bp deletions in the first exon respectively, for further analysis (Figure 4a; Figure S10). We observed that the seedling growth of *WOX11*‐KO lines was significantly slower than that of WT, and the shoot height, primary root length and crown root number of 7‐day‐old seedlings of *WOX11*‐KO and *slg2* were highly similar (Figure 3d–f), and the expression level of genes related to shoot and root development was also similarly decreased (Figure 3g,h). These observations were consistent well with the previous findings (Cheng *et al*., 2018; Jiang *et al*., 2017). In addition, we found that the overall morphology of *WOX11*‐KO adult plants resembled that of the *slg2* mutant (Figure 4b). Especially, the grain width of *WOX11*‐KO plants was reduced to a level similar to *slg2* (Figure 4c,e), while the grain length of these knockout plants remained unchanged (Figure 4d,e). These results indicate that *WOX11* also plays a role in the development of reproductive organs. We also performed cytological observation to understand the cellular processes mediated by *WOX11*, and found that the cell width and the cell area of spikelet hulls decreased similarly in the *WOX11*‐KO plants and the *slg2* mutant (Figure S11). We further analysed the expression of the cell expansion genes downregulated in the *slg2* mutant. As expected, transcription of all the six genes were greatly suppressed in the *WOX11*‐KO plants (Figure 4f). The physical interaction of the two proteins and the phenotypic similarity of the two null mutants strongly suggest that *SLG2* and *WOX11* act jointly to regulate grain width. To verify this speculation, we created the *slg2,wox11* double mutant by crossing the *slg2* mutant with the *WOX11*‐KO1 plants. We found that the grain width of the *slg2,wox11* double mutant was completely the same as the *slg2* and *WOX11*‐KO single mutants (Figure 4b–e). Consistently, the transcript levels of cell expansion genes were similarly downregulated in the *slg2,wox11* double mutant and the two single mutants (Figures 3g,h and 4f). Taken together, these results clearly demonstrate that *SLG2* functions coordinately with *WOX11* in grain width regulation.

### SLG2 acts as a transcription activator of WOX11

**Figure 5:**
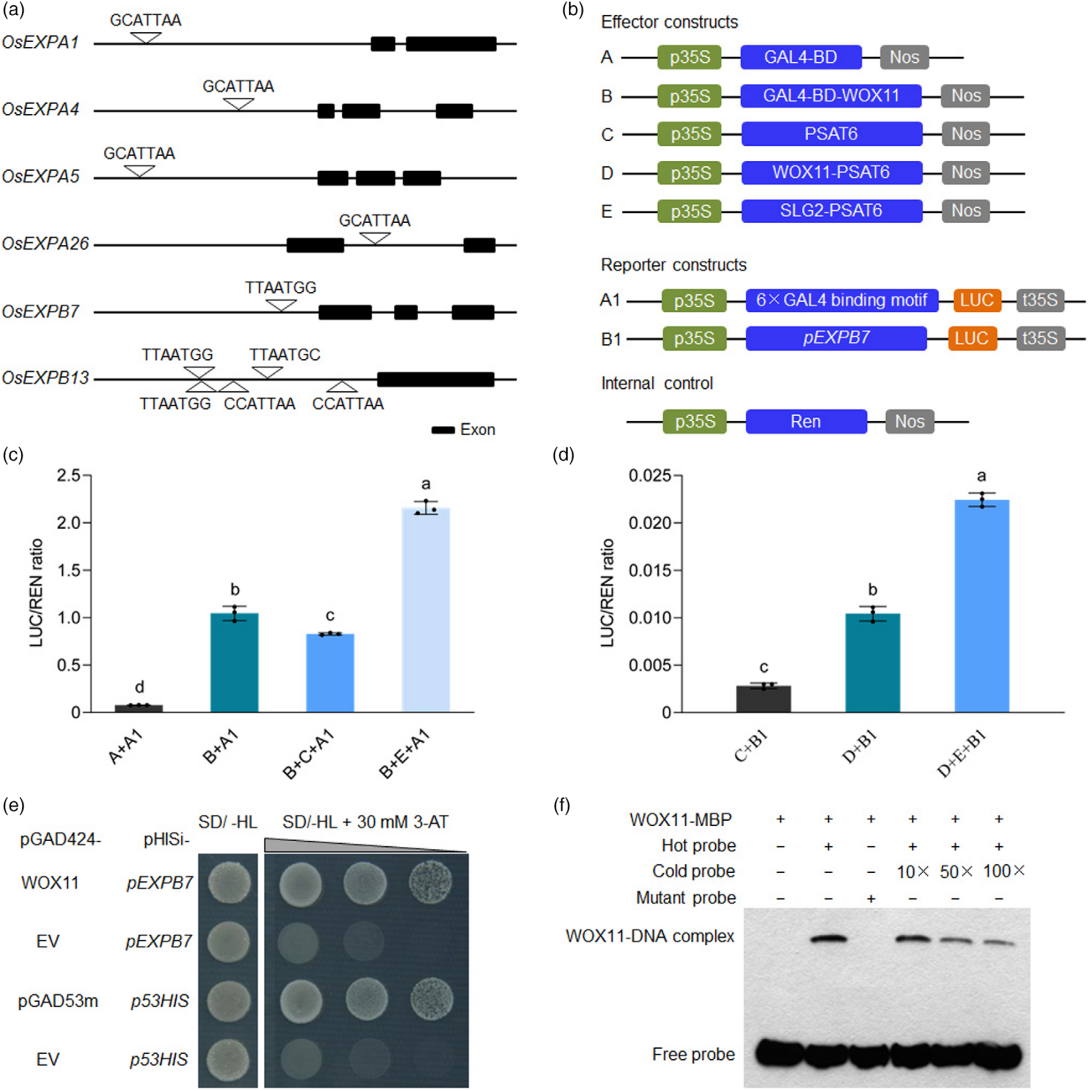
SLG2 functions as an activator of WOX11 to regulate the transcription of downstream cell expansion genes. (a) Scanning of the WOX11 binding motif ‘TTAATGG/C’ across the whole genomic sequence of the cell expansion genes downregulated in *slg2* and the *WOX11*‐KO lines. The length of promoter sequence is 2‐kb. (b) Schematic diagram of the constructs used for transactivation activity assay. (c) Analysis of the effect of SLG2 on the transactivation activity of WOX11. Data are means ± SD (*n* = 3). Bars followed by different letters represent significant difference at 5%. Indicating that SLG2 significantly enhances the transactivation activity of WOX11. (d) Transcription activation of WOX11 and SLG2 on *OsEXPB7*. Data are means ± SD (*n* = 3). Bars followed by different letters represent significant difference at 5%. Indicating the coordinate function of SLG2 and WOX11 in promoting the transcription of *OsEXPB7*. (e, f) WOX11 directly binds to the promoter of *OsEXPB7*, which is tested by yeast one‐hybrid assay (e) and electrophoretic mobility shift assay (EMSA) (f). The combinations pGAD53m‐*p53HIS* and pGAD424‐*p53HIS* are used as the positive and negative controls, respectively (e). In (f), the hot probe is a biotin‐labelled fragment of the *OsEXPB7* promoter sequence AGGGATCGATCGAAATTAATGGCGGGCAGGAGCAGGA, and the cold probe is a non‐labelled competitive probe. The mutant probe is the labelled hot probe sequence with two nucleotides mutated in the conserved binding site (AGGGATCGATCGAAATCCATGGCGGGCAGGAGCAGGA).

WOX11 has been shown to specifically bind to the WOX consensus ‘TTAATGG/C’ (Jiang *et al*., 2017). As described above, the transcription of cell expansion genes *OsEXPA1*, *OsEXPA4*, *OsEXPA5*, *OsEXPA26*, *OsEXPB7* and *OsEXPB13* was suppressed considerably in both *slg2* and *WOX11*‐KO plants (Figure 4f). To understand whether these six genes were the direct targets of WOX11, we first analysed the genomic sequences of these genes to see if there are any WOX11‐binding motifs. We found that all the six genes contain at least one conserved *cis*‐elements of TTAATGG/C in the 2‐kb promoter region or in the gene body (Figure 5a). Next, we explored the functional relationship between SLG2 and WOX11 by the dual‐luciferase reporter system in rice protoplasts (Figure 5b). We detected strong transactivation activity of WOX11 in our system (Figure 5c), and the activation activity of WOX11 was substantially enhanced by the SLG2 protein (Figure 5c). We further examined whether SLG2 and WOX11 jointly regulate the transcription of *OsEXPB7*, a potential downstream target showing the most severely suppressed transcription in the *WOX11*‐KO plants (Figure 4f). We found that WOX11 could effectively activate the transcription of *OsEXPB7*, and the combination of SLG2‐WOX11 further promoted the transcription of this gene (Figure 5d). Taken together, these results suggest that SLG2 acts as an activator of WOX11‐mediated transcriptional regulation.

To verify whether the SLG2‐WOX11 pathway directly regulates the expression of cell expansion genes, we tested the binding of WOX11 to the promoter of *OsEXPB7* by yeast one‐hybrid assay, where WOX11 was fused to GAL4 AD and the *OsEXPB7* promoter fused to the *HIS3* reporter gene. The result showed that WOX11 bound to the promoter, leading to activation of the *HIS3* reporter (Figure 5e). We further confirmed the binding of WOX11 to the *OsEXPB7* promoter by electrophoretic mobility shift assay (EMSA). We expressed the WOX11 protein in *E. coli* and purified it as a maltose binding protein (MBP)‐fusion protein. For the probes, we synthesized biotin‐labelled oligonucleotides containing the native (hot probe) and mutated (mutant probe) WOX binding site presented in the *OsEXPB7* promoter (Figure 5a and Table S1). As shown in Figure 5f, WOX11‐MBP bound strongly the hot probe but not the mutant probe. The specificity of the binding was further confirmed by including the cold probe as the competitor, where the competitor greatly reduced binding to the probe. Together, these results clearly show that the WOX11 protein binds to TTAATGG in the *OsEXPB7* promoter.

### The combination of *slg2* and *gw8* produces more slender grains

**Figure 6:**
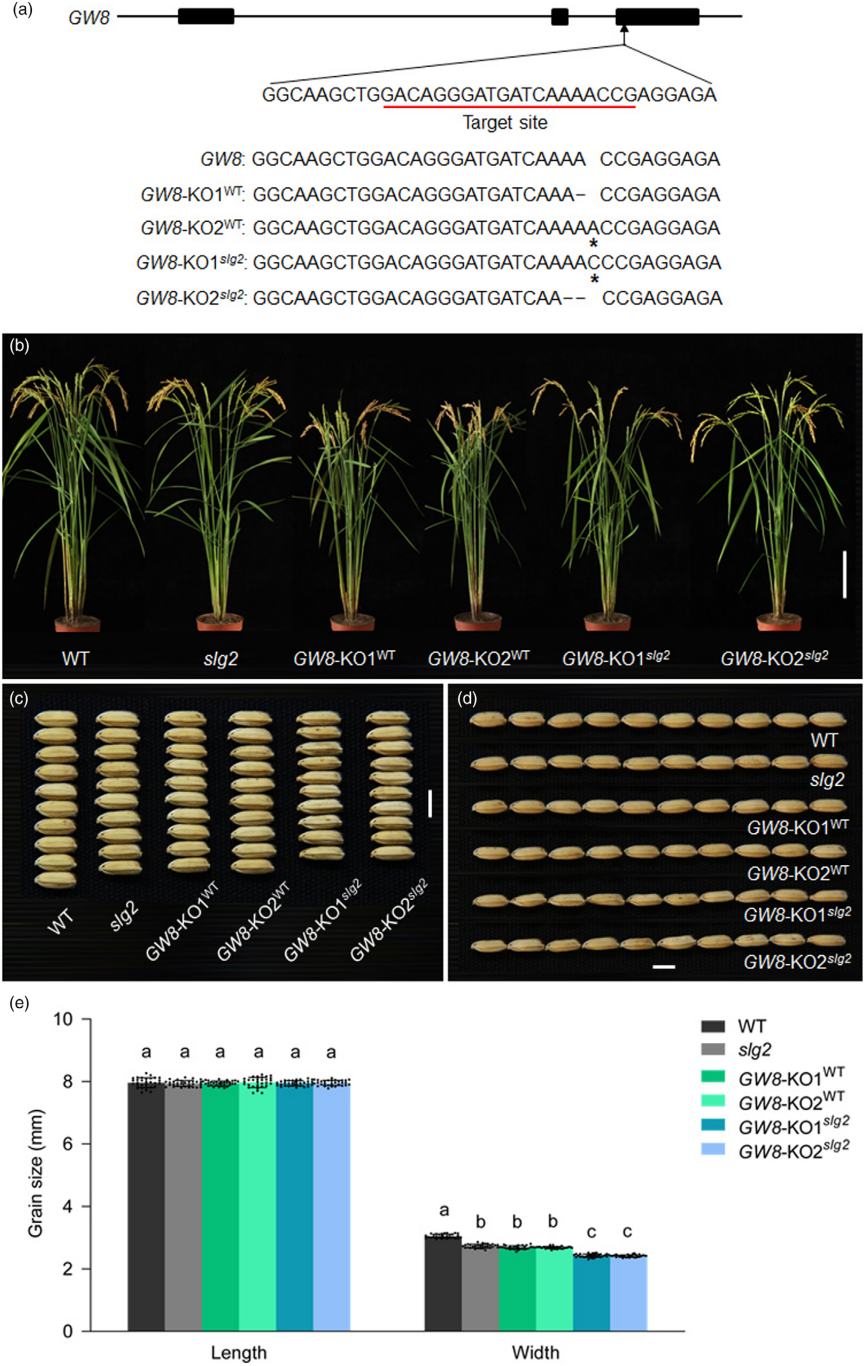
Fine regulation of grain width by *slg2*, *gw8* and their combination. (a) Creation of *GW8* knockout transgenic line by the CRISPR‐Cas9 genome editing system. The target site is red‐underlined. The representative transgenic lines (abbreviated as *GW8*‐KO1 and *GW8*‐KO2, respectively) are generated in ZY66 (*GW8*‐KO^WT^) and *slg2* (*GW8*‐KO^*slg2*^) genetic background. The asterisks indicate inserted nucleotides and the dashes indicate deleted nucleotides. (b) Plant morphology of WT, *slg2*, *GW8*‐KO^WT^ and *GW8*‐KO^*slg2*^ at the maturation stage. Bar = 15 cm. (c, d) Comparison of grain width (c) and grain length (d) among WT, *slg2*, *GW8*‐KO^WT^ and *GW8*‐KO^*slg2*^. Bars = 5 mm. (e) Statistical analysis of grain length and grain width in WT, *slg2*, *GW8*‐KO^WT^ and *GW8*‐KO^*slg2*^. Data are means ± SD (*n* = 30). Bars followed by different letters represent significant difference at 5%.

To carry out rational design of grain size, it is necessary not only to discover a sufficient number of functional genes, but also to understand the interacting effects between these genes (Lee *et al*., 2015; Sun *et al*., 2018; Zhong *et al*., 2020a,b). The latter should be more important in the flexible adjustment of the target trait to meet environment and/or human needs. Therefore, we tried to reveal the combined effect of *SLG2* and other grain width regulators by using CRISPR genome editing system, because this technology seems to be the most effective method in the current strategy of pyramiding favourable null alleles. To this end, we summarized the functions of the grain width‐related genes identified so far (Table S2), and selected the positive grain width regulator *GW8* (Wang *et al*., 2012). We knocked out *GW8* in the background of *slg2* (*GW8*‐KO^*slg2*^) and ZY66 (*GW8*‐KO^WT^) respectively, by specifically editing a target site in the third exon (Figure 6a). After sequencing the target region of the transgenic plants, we selected two homozygous *GW8*‐KO^WT^ lines with 1‐bp deletion and 1‐bp insertion in the coding region for phenotypic analysis (Figure 6a; Figure S12). We found that the obvious change in the overall morphology of the *GW8*‐KO^WT^ plants was the reduction of plant height, which was about 9.5% and 5.9% lower than WT and *slg2* respectively (Figure 6b; Figure S13a). Similar to the previous finding (Wang *et al*., 2012), we observed that the grain width of *GW8*‐KO^WT^ plants decreased significantly, even about 1.6% narrower than that of *slg2* (Figure 6c,e). It was reported that dysfunction of *GW8* decreases grain width but increases grain length (Wang *et al*., 2012). However, we did not find any change in the grain length of *GW8*‐KO^WT^ plants (Figure 6d,e), which may be due to the different genetic backgrounds of the receptor varieties. We also obtained two homozygous *GW8*‐KO^*slg2*^ lines with a C insertion and an AA deletion in the coding region (Figure 6a; Figure S12). We found that the *slg2,gw8* double mutants displayed overall morphology highly resembled the *slg2* single mutant, including plant height, panicle number, panicle size, and seed setting rate (Figure 6b; Figure S13). The only phenotypic difference between *slg2,gw8* and *slg2* was the grain size. The grain width of the double mutant was about 11.4% and 9.8% less than that of the single mutant *slg2* and *gw8*, respectively, while the grain length did not change significantly (Figure 6c–e). Notably, similar to the *slg2* mutant, the transparency of brown rice endosperm and the starch packaging in mature grains of *gw8* and *slg2,gw8* were completely normal (Figure S1). The reduction of grain width of the double mutants led to the decrease of 1000‐grain weight and thus grain yield per plant (Figure S13d,f). We also examined the expression of genes related to cell division and cell expansion in the spikelet hulls of the *slg2,gw8* plants, and found that the transcripts of all these genes were markedly reduced in the double mutant (Figure S14). This observation is consistent with the positive functions of *GW8* (Wang *et al*., 2012) and *SLG2* (Figure 1m; Figure S6e) in these cellular processes. Collectively, our results suggest that the additive effect of *slg2* and *gw8* alleles is limited to the decrease of grain width, which is of potential value in improving grain appearance quality.

## Discussion

### *SLG2* is a unique grain width regulator

Grain width as one of the key grain size determinants is particularly important for not only grain yield but also appearance quality, and the slender grain is more favoured by the consumers. So far, mutant analysis has identified several grain width‐related genes in rice, including *HGW* (Li *et al*., 2012), *GS6* (Sun *et al*., 2013), *OsARG* (Ma *et al*., 2013), *GW5L* (Tian *et al*., 2019), and *WG7* (Huang *et al*., 2020). Although loss of *GS6* function increases grain width (Sun *et al*., 2013), it is unclear whether manipulation of *GS6* expression could produce slender grains. Ectopic expression of *GW5L* effectively reduces grain width, but grain length of the transgenic plants also changes significantly (Tian *et al*., 2019). In contrast, dysfunction of *HGW*, *OsARG* and *WG7* specifically decreases the grain width. However, the null mutations of the three genes all lead to fatal defects in rice development, such as severe dwarfing and late heading in *hgw* (Li *et al*., 2012), low seed setting in *Osarg* (Ma *et al*., 2013), and severe dwarfing and low seed setting in *wg7* (Huang *et al*., 2020). On the other hand, extensive analysis of natural populations has isolated seven grain width‐related QTLs, including *GW2* (Song *et al*., 2007), *GW5* (Liu *et al*., 2017), *GS5* (Li *et al*., 2011), *GW8* (Wang *et al*., 2012), *GW6* (Shi *et al*., 2020), *TGW2* (Ruan *et al*., 2020), and *TGW12a* (Du *et al*., 2021). Although these genes have been reported to play a major role in regulating grain width, only four of them, *GS5*, *GW5*, *TGW2* and *TGW12a* appear to function exclusively in grain width determination (Table S2). Moreover, in addition to regulating the grain width, all the four genes also seem to play a role in the grain filling process (Du *et al*., 2021; Li *et al*., 2011; Ruan *et al*., 2020; Tian *et al*., 2019). Regretfully, knockout of *TGW12a* increases the formation of chalky endosperm. Although we are not clear whether knockout of *GW5* affects starch accumulation, compared with *GW5L*‐KO, *GW5*‐KO shows a wider grain but similar grain weight, which strongly suggest that *GW5*‐KO plants also face the problem of grain filling (Tian *et al*., 2019). Overexpression of *TGW2* effectively reduced the grain width, however, the negative effect of this gene on grain filling makes it uncertain whether the ectopic expression would reduce the grain filling rate and deteriorate the grain quality. Similarly, we are concerned about whether there is grain filling problem in the *gs5* mutant. In this study, we find that the loss of *SLG2* function leads to a significant decrease in grain width, without any effects on grain length, grain thickness, grain filling and grain quality (Figure 1a–e; Figures S1 and S2). Therefore, *SLG2* is a specific grain width regulator of potential value in improving rice appearance quality.

Our genetic analysis demonstrates that *SLG2* is one member of the WD40 superfamily (Figure 2). In rice, some *OsWD40s* have been revealed to function in specific developmental processes. For example, *GORI* is a seven WD40 repeats protein encoding gene, its knockout transgenic plants display male sterility, while these plants do not show any growth defects during the whole developmental stages (Kim *et al*., 2021). *OsTTG1* is a WD40 family gene that has been artificially selected during domestication. Loss of *OsTTG1* function significantly reduces anthocyanin accumulation in various rice organs with no adverse effects on their development (Yang *et al*., 2021). Recently, the WD40 protein gene *OsKRN2* has been found to present convergent selection in maize and rice (Chen *et al*., 2022). Knockout of *OsKRN2* greatly increases rice grain yield with no apparent trade‐offs in other agronomic traits. The rice functional genomics study on grain size regulation has uncovered hundreds of related genes. Surprisingly, almost none of these genes belong to the WD40 family, although the rice genome contains 231 WD40 protein genes (Yang *et al*., 2021). So far, mutant analysis has uncovered a role of several *OsWD40s* in grain development (Cheng *et al*., 2020; Gao *et al*., 2012; Nallamilli *et al*., 2013; Utsunomiya *et al*., 2011; Zhang et al., 2021a,b,c). However, all these mutants display severe defects in multiple important agronomic traits, such as extreme dwarf, low seed setting or preharvest sprouting. The growth defects of these mutants are in sharp contrast to *slg2*, which displays relatively normal overall phenotypes except the reduced panicle size and grain yield. Therefore, the grain width regulator *SLG2* represents a unique member of the WD40 family that could be applied in rice breeding. Further study would reveal whether there are WD40 family genes being artificially selected as grain size regulators during rice domestication.

### The *SLG2‐WOX11* module presents a novel pathway regulating grain width

The central role of WD40 repeats is to provide a platform for the interaction and assembly of several proteins into a signalosome (Jain and Pandey, 2018). Therefore, the WD40 proteins carry out their regulatory functions mainly by associating with other proteins. For example, OsWDR5a interacts with OsTrx1 to form the core components of the COMPASS‐like complex and regulates rice flowering and panicle branching (Jiang *et al*., 2018). OsTTG1 regulates anthocyanin biosynthesis by forming a MBW complex with Kala4, OsC1, OsDFR and Rc (Yang *et al*., 2021). The recently discovered OsKRN2 controls grain number by interacting with OsDUF1644, although the underlying mechanism of this interaction remains to be understood (Chen *et al*., 2022). In this study, we identify WOX11, a member of the WOX family protein, as the interactor of SLG2 (Figure 3a–c). As a central transcription factor, WOX11 has been revealed to function in root development of both rice and *Arabidopsis* (Sheng *et al*., 2017; Zhao *et al*., 2015), and regulates rice shoot development by recruiting a histone H3K27me3 demethylase JMJ705 (Cheng *et al*., 2018). Similar to the *WOX11*‐KO plants, the *slg2* mutant also presents defects in crown root and shoot development (Figure 3d), indicating that *SLG2* and *WOX11* jointly act on rice seedling development. Although the WOX family genes display various biological functions in plant growth regulation (Jha *et al*., 2020), there is still no information on the involvement of these genes in reproductive organ development. In this study, we reveal that the SLG2‐associated WOX11 is a grain width regulator (Figure 4; Figure S11). We demonstrate that WOX11 can activate the expression of downstream cell expansion genes by directly binding to the promoters of these genes, and the transactivation activity of WOX11 can be enhanced by SLG2 (Figure 5). In addition, we find that there is no obvious difference in grain width among the *slg2,wox11* double mutant, the *slg2* single mutant and the *WOX11*‐KO plants (Figure 4). These results suggest that *SLG2* is an essential component of *WOX11*‐mediated signalling pathways. Previous study has shown that WOX11 works as a transcription repressor of downstream *RR2* gene to regulate rice crown root development (Zhao *et al*., 2009). The differential functions of *WOX11* in grain width and crown root formation are highly similar to that of the *Arabidopsis* WOX family gene *WUS*, which acts as a bifunctional transcription factor: an inhibitor in stem cell regulation, and an activator in flower formation (Leibfried *et al*., 2005; Lohmann *et al*., 2001). And, the multifaceted functions of *WOX11* in various developmental processes should be attributed to its ability to interact with various cofactors, such as the interaction with ERF3 in crown root development (Zhao *et al*., 2015), the interaction with ADA2 in root meristem development (Zhou *et al*., 2017), and the interaction with JMJ705 in shoot meristem development (Cheng *et al*., 2018). In this study, we present evidence that WOX11 physically interacts with SLG2, both *in vivo* and *in vitro* (Figure 3a–c). The high phenotypic similarity of the two null mutants and the double mutant, the strong interaction between the two proteins, the transcriptional regulation of the same groups of downstream genes, together with the first discovery of *WOX11* involved in grain size regulation clearly indicate that the *SLG2‐WOX11* module presents a novel pathway to regulate grain width.

### The *SLG2‐GW8* combination shows great potential in designing grain width

Grain size is one of the most complex traits controlled by a large number of genes and their interactions (Jiang *et al*., 2022). So far, intensive efforts have been made to understand the molecular mechanism of grain size regulation, and exploration of the combinatory effects of several major genes on grain size also have been conducted in rice during the past decade (Lee *et al*., 2015; Ngangkham *et al*., 2018; Sun *et al*., 2018; Yang *et al*., 2023; Zhong *et al*., 2020a,b). However, most of the studies on the interaction of grain size‐related genes were carried out in natural populations. Due to the differences in genetic backgrounds, it is difficult to accurately evaluate the interacting effects of the allelic combinations. So far, there is only one report that describes the interaction of grain size genes under the same genetic background (Sun *et al*., 2018). At present, rice breeders are mainly committed to developing slender rice varieties, because it is preferred by rice consumers in many countries. Unfortunately, in most cases, the increase of grain length is often accompanied by the change of grain width, and increasing grain size to make it larger than medium would compromise fitness of the rice plant (Sun *et al*., 2018), such as grain number reduction, quality decline, lodging, and premature senescence. Therefore, moderate improvement of grain size is of extreme importance for the balanced plant development of comprehensive agronomic traits. In this study, we describe the combined effects of *SLG2* with *GW8* by the CRISPR/Cas9 genome editing system, because these two genes show positive effects on grain width by regulating different cellular processes, i.e., *GW8* regulates cell division and *SLG2* regulates cell expansion (Figure 1; Wang *et al*., 2012). We find that the regulatory effects of the two null alleles *slg2* and *gw8* on grain width differ to some extent, with *gw8* a stronger effect on grain width reduction. And, the additive effect of the two alleles produces more slender grains. Importantly, there is no obvious change in the grain length and starch accumulation of the double mutant and the two single mutants (Figure S1; Figure 6). Therefore, our results provide a promising strategy to fine tune the grain width of the receptor variety without changing other grain traits. That is, if the plant height of the receptor variety is appropriate, *slg2* or *slg2/gw8* combination would be a better choice to combine with the grain number genes. Alternatively, if the plant height of the receptor variety is somewhat high, the *gw8* allele could be applied to reduce both the grain width and the plant height. In other words, we could reduce the grain width of the receptor variety to varying degrees by applying the *slg2* and *gw8* alleles individually or in combination, and maintain/enhance the grain yield through pyramiding with the genes related to grain number and/or plant height, thus improving the appearance quality without reducing the yield.

In summary, we report the identification of *SLG2* as a novel and specific regulator of grain width in rice. We find that SLG2 recruits and activates WOX11 to directly promote the expression of downstream cell expansion genes. We provide a strategy to fine tune the grain width through the combination of *SLG2* with the grain width regulator *GW8*. Our results suggest that the *SLG2‐WOX11* module defines a novel pathway in rational design of rice plants with better grain appearance quality.

## Methods

### Plant materials and growth conditions

The *slg2* mutant was identified from the NaN_3_‐mutagenized M_2_ population of ZY66 (*japonica*). All the materials used in this study were cultivated in the experimental fields of the Institute of Genetics and Developmental Biology in Changping (40.2°N/116.2°E), Beijing during the summer or Lingshui (18.5°N, 110.0°E), Hainan province during the winter.

For seedling culture, healthy seeds were surface‐sterilized with 3% sodium hypochlorite for 30 min, soaked at 37 °C for 3 days. Germinating seeds were sowed into 96‐well PCR plates, and water‐cultured in the phytotron (SANYO) with 12 h light (28 °C)/12 h dark (28 °C), 65%–70% relative humidity, and 150 μM/m^2^/s photon flux density. Morphological investigation and RNA isolation were performed on 7‐day‐old seedlings.

### Map‐based cloning of *SLG2*

The mapping population was generated by crossing *slg2* with KY131 (*japonica*). Whole genome polymorphic markers were designed based on resequencing data of the two parents (30×), and primers used for fine mapping are listed in Table S1. Using 20 bulked F_2_ plants with WT and mutant phenotypes respectively, the candidate gene was first mapped to the short arm of chromosome 2 between the markers M1 and M2. Further analysis of the F_2_ mutant plants subsequently fine‐mapped the causal gene to the region between the markers M6 and M7, and sequence comparison was then performed between *slg2* and WT based on the resequencing data (30×).

### Morphological and histological analysis

Investigation of agronomic traits was performed on plant height, days to heading, panicle number, grain number per main panicle, 1000‐grain weight, seed setting rate and grain yield per plant at the maturation stage. Caryopsis development was observed on florets marked at the time of anthesis, and developing ovaries were collected at different developmental stages. The grain filling rate was calculated as the percentage of the dry grain weight at the two adjacent indicated days. The length, width and thickness of mature grains were measured using Olympus stream software under a light microscope (SZX16; Olympus, Japan).

For histological analysis, florets before anthesis were sampled and fixed in formalin‐acetic acid alcohol (FAA) solution (50% ethanol, 5% acetic acid and 3.7% formaldehyde) overnight at 4 °C after 20 min vacuum treatment. After dehydration with a gradient of ethanol, replacement with xylene and embedding in paraffin (8002‐74‐2, Sigma‐Aldrich, Steinheim, Germany), sections (8 μm thick) were cut with a rotary microtome (RM2235, LEICA, Heidelberger, Germany), stained with 0.2% toluidine blue and observed under a microscope (BX53; Olympus). The outer parenchyma cell number and cell area were measured with Olympus stream and Image J software, respectively. For SEM observation, the outer glumes of fully mature, dry grains were sprayed with gold particles and scanned with a scanning electron microscope (S‐3000N; Hitachi, Tokyo, Japan). The outer glume cell number was counted and cell size was measured using the Olympus stream software (SZX16; Olympus, Japan). To observe the grain endosperm structure, mature grains were hand‐cut using the bladeless cutter, then the cutting surfaces were coated with gold in a vacuum with an ion sputtering device (JFC‐1100E; JEOL, Tokyo, Japan), and observed with a scanning electron microscope (S‐3000N; Hitachi, Japan).

### RNA isolation and RT‐qPCR analysis

Samples were taken from shoots and roots of 7‐day‐old seedlings, and from flag leaf blade, flag leaf sheath, culm, young panicle and mature floret before anthesis. Total RNA was extracted using RNAiso PLUS reagent (Takara, Dojima, Japan), and 1 μg RNA was reverse‐transcribed by oligo (dT) primers using a reverse transcription kit (Promega, Madison, WI, USA) after digestion with RNase‐free DNaseI (Thermo Scientific, Vilnius, Lithuania). The RT‐qPCR assay was performed in triplicate with SYBR Green I Master reagent and the Light Cycler Nano system (Roche, Mannheim, Germany). *OsActin* was used as the internal control for normalization. The primers used are listed in Table S1.

### Vector construction and transformation

For complementation test, a 17.8‐kb genomic fragment (containing the entire coding region, 2392‐bp upstream of ATG and 1567‐bp downstream of TGA) was amplified into four fragments, and cloned into the pZH2B vector by a seamless cloning kit (CloneSmarter, Fort Bend County, TX). For knockout vector, a 20‐bp carefully designed target was selected from the third exon of *SLG2*, the first exon of *WOX11* and the third exon of *GW8* by the CRISPR‐GE genome editing tool (http://skl.scau.edu.cn/) and ligated to the CRISPR/Cas9 vector pHUN4C12 (Xie *et al*., 2017). For overexpression construct, the coding sequence of *SLG2* was amplified from ZY66 and cloned into the pZH2Bi vector driven by the ubiquitin promoter. These vectors were transformed into *slg2* with the *Agrobacterium tumefaciens*‐mediated transformation method. For the knockout assay, the T_1_ plants without exogenous DNA were selected, and the target sites were sequenced. From these plants, the homozygous mutants were obtained, and the offspring of these homozygous lines were used for phenotypic analysis. The primers used for vector construction and transformant selection are listed in Table S1.

### Yeast two‐hybrid assay

The coding sequences of *SLG2* and *WOX11* were cloned into the pGADT7 or pGBKT7 vector (Clontech, Mountain View, CA, USA), and the resulting constructs and the corresponding empty vectors were then co‐transformed into the yeast strain Golden Yeast with different combinations. Interactions were detected on SD/−Leu‐Trp‐His‐Ade medium. The transformation was conducted according to the Yeast Two‐Hybrid System User Manual (Clontech). The primers used in this assay are listed in Table S1.

### Transient expression assay in rice protoplast

For subcellular localization, the coding sequence of *SLG2* was inserted into the pSAT6‐EYFP‐N1 vector (PSAT6) to construct the *SLG2‐EYFP* (*SLG2‐PSAT6*) vector. The empty vector was used as a negative control. For the BiFC assay, the coding sequences of *SLG2* and *WOX11* were ligated into the pUC19‐VYNE (R) or pUC19‐VYCE (R) vector fused with the N‐ or C‐terminus of the Venus YFP sequence, respectively. These plasmids and the corresponding empty vectors were co‐transformed in different combinations into rice protoplasts. The fluorescent signal was detected with a confocal laser scanning microscope (Leica TCS SP5) after 28 °C incubation for 16 h in the dark.

For transactivation activity, the coding sequence of *WOX11* was fused with the GAL4 DNA binding domain in the pRT‐BD vector, and the resulting construct (GAL4‐BD‐WOX11) was used as the effectors. The coding sequence of *SLG2* was fused with pSAT6‐EYFP‐N1 vector to form SLG2‐PSAT6 fusion protein, and was taken as the cofactor of WOX11. The PSAT6 protein was used as the control. The LUC vector, which contains six copies of GAL4 binding motif and luciferase coding region, was used as the reporter, and the vector that expressed *Renilla* luciferase (pTRL) was employed as the internal control. The effectors and cofactors were then co‐transformed with the reporter and the internal control into rice protoplasts.

To detect the WOX11‐mediated activation of *OsEXPB7* expression, the coding sequence of *WOX11* was fused with PSAT6 (WOX11‐PSAT6) as the effector, and the promoter sequence of *OsEXPB7* was cloned into the LUC vector by replacing the 35S promoter (*pEXPB7*‐*LUC*) and acted as the reporter. The above‐mentioned fused protein SLG2‐PSAT6 was used as the cofactor of WOX11, and the PSAT6 empty vector was used as the negative control. As the internal controls, the LUC and pTRL vectors were co‐transformed with the resulting constructs into rice protoplasts. All transformations were performed via the PEG (polyethylene glycol) mediated method. After 16 h incubation at 28 °C in the dark, the protoplast was centrifuged at 150 ***g*** and the pellet was used for the dual‐luciferase assay as described in the Dual‐Luciferase® Reporter Assay System Manual (Promega), and the relative LUC/REN ratio was measured with a luminometer (GLOMAX; Promega). Primers used for these assays are listed in Table S1.

### Pull‐down assay

The coding sequence of *SLG2* was inserted into the pGEX‐4T‐1 vector to express GST‐SLG2, and the coding sequence of *WOX11* was inserted into the pMAL‐c5X vector to express MBP‐WOX11. All plasmids were transformed into *Escherichia coli* strain BL21. Fusion proteins were induced with 1.0 mM IPTG at 37 °C for 5 h. Anti‐GST (1:5000; Proteintech, Wuhan, China) and anti‐MBP (1:2000; Proteintech, America) antibodies were used in immunoblotting analysis. GST pull‐down assay was performed as previously described (Zhang et al., 2021a,b,c). Primers used for this assay are listed in Table S1.

### Yeast one‐hybrid assay

The coding sequence of *WOX11* was fused with the GAL4 activation domain of pGAD424 (Clontech), forming pGAD424‐WOX11. To generate *pEXPB7‐pHISi* reporter vector, the promoter fragment of *OsEXPB7* was synthesized and cloned into the pHISi vector. The plasmids were co‐transformed into yeast strain YM4271, and DNA‐protein interactions were determined by the growth of the transformants on the nutrient‐deficient medium with 30 mM 3‐amino‐1,2,4‐triazole (3‐AT), following the manufacturer's manual (Clontech). Primers used in this assay are listed in Table S1.

### Electrophoretic mobility shift assay (EMSA)

The LightShift Chemiluminescent EMSA Kit (No. 20148; ThermoFisher SCIENTIFIC, America) was used in this experiment. Biotin was labelled at the 5′ end of *cis*‐element. The biotin‐labelled DNA was synthesized by RuiBiotech (Beijing, China). The WOX11‐MBP fusion protein was expressed in *Escherichia coli* strain BL21 and purified using amylose resin (BioLabs, America) affinity chromatography. The detailed procedure of EMSA follows the manufacturer's instructions. Photos were taken using Charge‐coupled device (CCD) camera. Primers and probe sequences used for EMSA are listed in Table S1.

### Protein sequence alignment

The protein sequence of SLG2 and HSLG2 were downloaded from Ensembl Plants (http://plants.ensembl.org/index.html), and alignments were performed using the online Clustal Omega program (https://www.ebi.ac.uk/Tools/msa/clustalo/) with manual curation.

### Accession numbers

Sequence data from this article can be found in the EMBL/GenBank data libraries under the following accession numbers: *SLG2*, *Os02g18820*; *WOX11*, *Os07g48560*; *HSLG2*, *Os09g12550*; *OsActin*, *Os03g50885*; *H1*, *Os04g18090*; *E2F2*, *Os12g06200*; *CAK1*, *Os06g07480*; *CDKA1*, *Os03g01850*; *MCM3*, *Os05g39850*; *CYCA2.1*, *Os12g39210*; *CYCB2.1*, *Os08g40170*; *CYCB2.2*, *Os06g51110*; *CDKB*, *Os05g40540*; *OsEXPA1*, *Os04g15840*; *OsEXPA2*, *Os01g60770*; *OsEXPA4*, *Os05g39990*; *OsEXPA5*, *Os02g51040*; *OsEXPA7*, *Os03g60720*; *OsEXPA10*, *Os04g49410*; *OsEXPA13*, *Os02g16730*; *OsEXPA16*, *Os06g41700*; *OsEXPA25*, *Os03g06010*; *OsEXPA26*, *Os12g36040*; *OsEXPA29*, *Os06g50400*; *OsEXPB3*, *Os10g40720*; *OsEXPB4*, *Os10g40730*; *OsEXPB6*, *Os10g40700*; *OsEXPB7*, *Os03g01270*; *OsEXPB12*, *Os03g44290*; *OsEXPB13*, *Os03g01650*; *GS5*, *Os05g06660*; *GS6*, *Os06g03710*; *GW2*, *Os02g14720*; *GW5*, *Os05g09520*; *GW5L*, *Os01g09470*; *GW6*, *Os06g15620*; *GW8*, *Os08g41940*; *HGW*, *Os06g06530*; *OsARG*, *Os04g01590*; *TGW2*, *Os02g52550*; *TGW12a*, *Os12g36660*; *WG7*, *Os07g47360*.

## Conflict of interest

The authors declare no conflict of interest.

## Author contributions

S.Y. conceived and supervised the project. D.X. performed the experiments, analysed the data and prepared the original draft. S.Y. designed the experiments and revised the manuscript. R.W. screened the *slg2* mutant, created the mapping population and carried out field management. Y.W. contributed to the reagents and equipment management. Y.L. and G.S. assisted in the data collection.

## Supporting information


**Figure S1** Observation of brown rice transparency (a) and starch packaging (b) from the mature grains of WT, *slg2*, *GW8*‐KO^WT^ and *GW8*‐KO^*slg2*^.
**Figure S2** *slg2* shows normal grain filling.
**Figure S3** Expression analysis of cell division and cell expansion genes (a) and grain width‐related genes (b) in *slg2* and WT.
**Figure S4** Comparison of agronomic traits between *slg2* and WT.
**Figure S5** The reduced grain width of *slg2* is caused by decreased cell expansion.
**Figure S6** Knockout of *SLG2* results in plants with the *slg2* mutant phenotype.
**Figure S7** Overexpression of *SLG2* fully rescues the *slg2* mutant phenotype.
**Figure S8** Protein sequence comparison between SLG2 and HSLG2.
**Figure S9** The *SLG2* homologue *HSLG2* shows no regulatory roles in rice development.
**Figure S10** Verification of the WOX11 knockout lines (*WOX11*‐KO) by PCR‐based sequencing.
**Figure S11** The *WOX11‐*KO lines show reduced grain width similar to the *slg2* mutant.
**Figure S12** Verification of the *GW8* knockout lines in the background of WT (*GW8*‐KO^WT^) and *slg2* (*GW8*‐KO^*slg2*^) by PCR‐based sequencing.
**Figure S13** Statistical analysis of plant height (a), panicle number (b), grain number per main panicle (c), 1000‐grain weight (d), seed setting rate (e), and grain yield per plant (f) of WT, *slg2* and *GW8*‐KO.
**Figure S14** Expression analysis of cell division and cell expansion genes in WT, *slg2*, *GW8*‐KO^WT^ and *GW8*‐KO^*slg2*^.

**Table S1** Primers used in this study.
**Table S2** Effects of *SLG2* and known grain width genes on four grain traits.

## References

[pbi14102-bib-0001] Chen, K. , Łyskowski, A. , Jaremko, Ł. and Jaremko, M. (2021) Genetic and molecular factors determining grain weight in rice. Front. Plant Sci. 12, 605799. 34322138 10.3389/fpls.2021.605799 PMC8313227

[pbi14102-bib-0002] Chen, W. , Chen, L. , Zhang, X. , Yang, N. , Guo, J. , Wang, M. , Ji, S. *et al*. (2022) Convergent selection of a WD40 protein that enhances grain yield in maize and rice. Science, 375, eabg7985. 35324310 10.1126/science.abg7985

[pbi14102-bib-0003] Cheng, S. , Tan, F. , Lu, Y. , Liu, X. , Li, T. , Yuan, W. , Zhao, Y. *et al*. (2018) WOX11 recruits a histone H3K27me3 demethylase to promote gene expression during shoot development in rice. Nucleic Acids Res. 46, 2356–2369. 29361035 10.1093/nar/gky017 PMC5861455

[pbi14102-bib-0004] Cheng, X. , Pan, M. , Zhiguo, E. , Zhou, Y. , Niu, B. and Chen, C. (2020) Functional divergence of two duplicated *Fertilization Independent Endosperm* genes in rice with respect to seed development. Plant J. 104, 124–137. 33463824 10.1111/tpj.14911

[pbi14102-bib-0005] Choi, B.S. , Kim, Y.J. , Markkandan, K. , Koo, Y.J. , Song, J.T. and Seo, H.S. (2018) GW2 functions as an E3 ubiquitin ligase for rice expansin‐like 1. Int. J. Mol. Sci. 19, 1904. 29958473 10.3390/ijms19071904 PMC6073362

[pbi14102-bib-0006] Dhatt, B.K. , Paul, P. , Sandhu, J. , Hussain, W. , Irvin, L. , Zhu, F. , Adviento‐Borbe, M.A. *et al*. (2021) Allelic variation in rice *Fertilization Independent Endosperm 1* contributes to grain width under high night temperature stress. New Phytol. 229, 335–350. 32858766 10.1111/nph.16897 PMC7756756

[pbi14102-bib-0007] Du, Z. , Huang, Z. , Li, J. , Bao, J. , Tu, H. , Zeng, C. , Wu, Z. *et al*. (2021) *qTGW12a*, a naturally varying QTL, regulates grain weight in rice. Theor. Appl. Genet. 134, 2767–2776. 34021769 10.1007/s00122-021-03857-4 PMC8354980

[pbi14102-bib-0008] Duan, P. , Rao, Y. , Zeng, D. , Yang, Y. , Xu, R. , Zhang, B. , Dong, G. *et al*. (2014) *SMALL GRAIN 1*, which encodes a mitogen‐activated protein kinase kinase 4, influences grain size in rice. Plant J. 77, 547–557. 24320692 10.1111/tpj.12405

[pbi14102-bib-0009] Duan, P. , Ni, S. , Wang, J. , Zhang, B. , Xu, R. , Wang, Y. , Chen, H. *et al*. (2015) Regulation of *OsGRF4* by OsmiR396 controls grain size and yield in rice. Nat Plants, 2, 15203. 27250749 10.1038/nplants.2015.203

[pbi14102-bib-0010] Fan, C. , Xing, Y. , Mao, H. , Lu, T. , Han, B. , Xu, C. , Li, X. *et al*. (2006) *GS3*, a major QTL for grain length and weight and minor QTL for grain width and thickness in rice, encodes a putative transmembrane protein. Theor. Appl. Genet. 112, 1164–1171. 16453132 10.1007/s00122-006-0218-1

[pbi14102-bib-0011] Gao, X. , Chen, Z. , Zhang, J. , Li, X. , Chen, G. , Li, X. and Wu, C. (2012) *OsLIS‐L1* encoding a lissencephaly type‐1‐like protein with WD40 repeats is required for plant height and male gametophyte formation in rice. Planta, 235, 713–727. 22020753 10.1007/s00425-011-1532-7

[pbi14102-bib-0012] Hu, J. , Wang, Y. , Fang, Y. , Zeng, L. , Xu, J. , Yu, H. , Shi, Z. *et al*. (2015) A rare allele of *GS2* enhances grain size and grain yield in rice. Mol. Plant, 8, 1455–1465. 26187814 10.1016/j.molp.2015.07.002

[pbi14102-bib-0013] Huang, K. , Wang, D. , Duan, P. , Zhang, B. , Xu, R. , Li, N. and Li, Y. (2017) *WIDE AND THICK GRAIN 1*, which encodes an otubain‐like protease with deubiquitination activity, influences grain size and shape in rice. Plant J. 91, 849–860. 28621888 10.1111/tpj.13613

[pbi14102-bib-0014] Huang, Y. , Bai, X. , Cheng, N. , Xiao, J. , Li, X. and Xing, Y. (2020) *Wide Grain 7* increases grain width by enhancing H3K4me3 enrichment in the *OsMADS1* promoter in rice (*Oryza sativa* L.). Plant J. 102, 517–528. 31830332 10.1111/tpj.14646

[pbi14102-bib-0015] Jain, B.P. and Pandey, S. (2018) WD40 repeat proteins: signalling scaffold with diverse functions. Protein J. 37, 391–406. 30069656 10.1007/s10930-018-9785-7

[pbi14102-bib-0017] Jha, P. , Ochatt, S.J. and Kumar, V. (2020) *WUSCHEL*: a master regulator in plant growth signaling. Plant Cell Rep. 39, 431–444. 31984435 10.1007/s00299-020-02511-5

[pbi14102-bib-0018] Jiang, W. , Zhou, S. , Zhang, Q. , Song, H. , Zhou, D.‐X. and Zhao, Y. (2017) Transcriptional regulatory network of *WOX11* is involved in the control of crown root development, cytokinin signals, and redox in rice. J. Exp. Bot. 68, 2787–2798. 28830102 10.1093/jxb/erx153 PMC5853245

[pbi14102-bib-0019] Jiang, P. , Wang, S. , Jiang, H. , Cheng, B. , Wu, K. and Ding, Y. (2018) The COMPASS‐Like complex promotes flowering and panicle branching in rice. Plant Physiol. 176, 2761–2771. 29440594 10.1104/pp.17.01749 PMC5884598

[pbi14102-bib-0020] Jiang, H. , Zhang, A. , Liu, X. and Chen, J. (2022) Grain size associated genes and the molecular regulatory mechanism in rice. Int. J. Mol. Sci. 23, 3169. 35328589 10.3390/ijms23063169 PMC8953112

[pbi14102-bib-0021] Jiao, Y. , Wang, Y. , Xue, D. , Wang, J. , Yan, M. , Liu, G. , Dong, G. *et al*. (2010) Regulation of *OsSPL14* by OsmiR156 defines ideal plant architecture in rice. Nat. Genet. 42, 541–544. 20495565 10.1038/ng.591

[pbi14102-bib-0023] Kim, Y.‐J. , Kim, M.‐H. , Hong, W.‐J. , Moon, S. , Kim, E.‐J. , Silva, J. , Lee, J. *et al*. (2021) *GORI*, encoding the WD40 domain protein, is required for pollen tube germination and elongation in rice. Plant J. 105, 1645–1664. 33345419 10.1111/tpj.15139

[pbi14102-bib-0024] Lee, C.‐M. , Park, J. , Kim, B. , Seo, J. , Lee, G. , Jang, S. and Koh, H.‐J. (2015) Influence of multi‐gene allele combinations on grain size of rice and development of a regression equation model to predict grain parameters. Rice, 8, 33. 26519289 10.1186/s12284-015-0066-1 PMC4627975

[pbi14102-bib-0025] Leibfried, A. , To, J.P.C. , Busch, W. , Stehling, S. , Kehle, A. , Demar, M. , Kieber, J.J. *et al*. (2005) WUSCHEL controls meristem function by direct regulation of cytokinin‐inducible response regulators. Nature, 438, 1172–1175. 16372013 10.1038/nature04270

[pbi14102-bib-0026] Li, Y. , Fan, C. , Xing, Y. , Jiang, Y. , Luo, L. , Sun, L. , Shao, D. *et al*. (2011) Natural variation in *GS5* plays an important role in regulating grain size and yield in rice. Nat. Genet. 43, 1266–1269. 22019783 10.1038/ng.977

[pbi14102-bib-0027] Li, J. , Chu, H. , Zhang, Y. , Mou, T. , Wu, C. , Zhang, Q. and Xu, J. (2012) The rice *HGW* gene encodes a ubiquitin‐associated (UBA) domain protein that regulates heading date and grain weight. PLoS One, 7, e34231. 22457828 10.1371/journal.pone.0034231 PMC3311617

[pbi14102-bib-0028] Li, N. , Xu, R. , Duan, P. and Li, Y. (2018) Control of grain size in rice. Plant Reprod. 31, 237–251. 29523952 10.1007/s00497-018-0333-6

[pbi14102-bib-0030] Liu, L. , Tong, H. , Xiao, Y. , Che, R. , Xu, F. , Hu, B. , Liang, C. *et al*. (2015a) Activation of *Big Grain1* significantly improves grain size by regulating auxin transport in rice. Proc. Natl Acad. Sci. USA, 112, 11102–11107. 26283354 10.1073/pnas.1512748112 PMC4568269

[pbi14102-bib-0031] Liu, S. , Hua, L. , Dong, S. , Chen, H. , Zhu, X. , Jiang, J.E. , Zhang, F. *et al*. (2015b) OsMAPK6, a mitogen‐activated protein kinase, influences rice grain size and biomass production. Plant J. 84, 672–681. 26366992 10.1111/tpj.13025

[pbi14102-bib-0032] Liu, J. , Chen, J. , Zheng, X. , Wu, F. , Lin, Q. , Heng, Y. , Tian, P. *et al*. (2017) *GW5* acts in the brassinosteroid signalling pathway to regulate grain width and weight in rice. Nat. Plants, 3, 17043. 28394310 10.1038/nplants.2017.43

[pbi14102-bib-0033] Lohmann, J.U. , Hong, R.L. , Hobe, M. , Busch, M.A. , Parcy, F. , Simon, R. and Weigel, D. (2001) A molecular link between stem cell regulation and floral patterning in *Arabidopsis* . Cell 105, 793–803. 11440721 10.1016/s0092-8674(01)00384-1

[pbi14102-bib-0034] Ma, X. , Cheng, Z. , Qin, R. , Qiu, Y. , Heng, Y. , Yang, H. , Ren, Y. *et al*. (2013) *OsARG* encodes an arginase that plays critical roles in panicle development and grain production in rice. Plant J. 73, 190–200. 26011250 10.1111/j.1365-313x.2012.05122.x

[pbi14102-bib-0035] Miao, J. , Yang, Z. , Zhang, D. , Wang, Y. , Xu, M. , Zhou, L. , Wang, J. *et al*. (2019) Mutation of *RGG2*, which encodes a type B heterotrimeric G protein γ subunit, increases grain size and yield production in rice. Plant Biotechnol. J. 17, 650–664. 30160362 10.1111/pbi.13005 PMC6381795

[pbi14102-bib-0036] Nallamilli, B.R.R. , Zhang, J. , Mujahid, H. , Malone, B.M. , Bridges, S.M. and Peng, Z. (2013) Polycomb group gene *OsFIE2* regulates rice (*Oryza sativa*) seed development and grain filling via a mechanism distinct from *Arabidopsis* . PLoS Genet. 9, e1003322. 23505380 10.1371/journal.pgen.1003322 PMC3591265

[pbi14102-bib-0037] Ngangkham, U. , Samantaray, S. , Yadav, M.K. , Kumar, A. , Chidambaranathan, P. and Katara, J.L. (2018) Effect of multiple allelic combinations of genes on regulating grain size in rice. PLoS One, 13, e0190684. 29304121 10.1371/journal.pone.0190684 PMC5755915

[pbi14102-bib-0039] Qin, X. , Huang, Q. , Xiao, H. , Zhang, Q. , Ni, C. , Xu, Y. , Liu, G. *et al*. (2016) The rice DUF1620‐containing and WD40‐like repeat protein is required for the assembly of the restoration of fertility complex. New Phytol. 210, 934–945. 26781807 10.1111/nph.13824

[pbi14102-bib-0040] Ruan, B. , Shang, L. , Zhang, B. , Hu, J. , Wang, Y. , Lin, H. , Zhang, A. *et al*. (2020) Natural variation in the promoter of *TGW2* determines grain width and weight in rice. New Phytol. 227, 629–640. 32167575 10.1111/nph.16540

[pbi14102-bib-0041] Sheng, L. , Hu, X. , Du, Y. , Zhang, G. , Huang, H. , Scheres, B. and Xu, L. (2017) Non‐canonical *WOX11*‐mediated root branching contributes to plasticity in Arabidopsis root system architecture. Development, 144, 3126–3133. 28743799 10.1242/dev.152132 PMC5611959

[pbi14102-bib-0042] Shi, C. , Ren, Y. , Liu, L. , Wang, F. , Zhang, H. , Tian, P. , Pan, T. *et al*. (2019) Ubiquitin specific protease 15 has an important role in regulating grain width and size in rice. Plant Physiol. 180, 381–391. 30796160 10.1104/pp.19.00065 PMC6501108

[pbi14102-bib-0043] Shi, C.‐L. , Dong, N.‐Q. , Guo, T. , Ye, W.‐W. , Shan, J.‐X. and Lin, H.‐X. (2020) A quantitative trait locus *GW6* controls rice grain size and yield through the gibberellin pathway. Plant J. 103, 1174–1188. 32365409 10.1111/tpj.14793

[pbi14102-bib-0044] Si, L. , Chen, J. , Huang, X. , Gong, H. , Luo, J. , Hou, Q. , Zhou, T. *et al*. (2016) *OsSPL13* controls grain size in cultivated rice. Nat. Genet. 48, 447–456. 26950093 10.1038/ng.3518

[pbi14102-bib-0045] Song, X.‐J. , Huang, W. , Shi, M. , Zhu, M.‐Z. and Lin, H.‐X. (2007) A QTL for rice grain width and weight encodes a previously unknown RING‐type E3 ubiquitin ligase. Nat. Genet. 39, 623–630. 17417637 10.1038/ng2014

[pbi14102-bib-0046] Stirnimann, C.U. , Petsalaki, E. , Russell, R.B. and Müller, C.W. (2010) WD40 proteins propel cellular networks. Trends Biochem. Sci. 35, 565–574. 20451393 10.1016/j.tibs.2010.04.003

[pbi14102-bib-0047] Sun, L. , Li, X. , Fu, Y. , Zhu, Z. , Tan, L. , Liu, F. , Sun, X. *et al*. (2013) *GS6*, a member of the GRAS gene family, negatively regulates grain size in rice. J. Integr. Plant Biol. 55, 938–949. 23650998 10.1111/jipb.12062

[pbi14102-bib-0048] Sun, S. , Wang, L. , Mao, H. , Shao, L. , Li, X. , Xiao, J. , Ouyang, Y. *et al*. (2018) A G‐protein pathway determines grain size in rice. Nat. Commun. 9, 851. 29487318 10.1038/s41467-018-03141-y PMC5829277

[pbi14102-bib-0049] Sun, W. , Xu, X.H. , Li, Y. , Xie, L. , He, Y. , Li, W. , Lu, X. *et al*. (2020) OsmiR530 acts downstream of OsPIL15 to regulate grain yield in rice. New Phytol. 226, 823–837. 31883119 10.1111/nph.16399 PMC7187366

[pbi14102-bib-0050] Tian, P. , Liu, J. , Mou, C. , Shi, C. , Zhang, H. , Zhao, Z. , Lin, Q. *et al*. (2019) *GW5‐Like*, a homolog of *GW5*, negatively regulates grain width, weight and salt resistance in rice. J. Integr. Plant Biol. 61, 1171–1185. 30450718 10.1111/jipb.12745

[pbi14102-bib-0051] Utsunomiya, Y. , Samejima, C. , Takayanagi, Y. , Izawa, Y. , Yoshida, T. , Sawada, Y. , Fujisawa, Y. *et al*. (2011) Suppression of the rice heterotrimeric G protein β‐subunit gene, *RGB1*, causes dwarfism and browning of internodes and lamina joint regions. Plant J. 67, 907–916. 21585570 10.1111/j.1365-313X.2011.04643.x

[pbi14102-bib-0052] Wang, S. , Wu, K. , Yuan, Q. , Liu, X. , Liu, Z. , Lin, X. , Zeng, R. *et al*. (2012) Control of grain size, shape and quality by *OsSPL16* in rice. Nat. Genet. 44, 950–954. 22729225 10.1038/ng.2327

[pbi14102-bib-0053] Wang, S. , Li, S. , Liu, Q. , Wu, K. , Zhang, J. , Wang, S. , Wang, Y. *et al*. (2015) The *OsSPL16‐GW7* regulatory module determines grain shape and simultaneously improves rice yield and grain quality. Nat. Genet. 47, 949–954. 26147620 10.1038/ng.3352

[pbi14102-bib-0054] Xie, X. , Ma, X. , Zhu, Q. , Zeng, D. , Li, G. and Liu, Y.‐G. (2017) CRISPR‐GE: a convenient software toolkit for crispr‐based genome editing. Mol. Plant, 10, 1246–1249. 28624544 10.1016/j.molp.2017.06.004

[pbi14102-bib-0055] Xu, H. , Zhao, M. , Zhang, Q. , Xu, Z. and Xu, Q. (2016) The *DENSE AND ERECT PANICLE 1* (*DEP1*) gene offering the potential in the breeding of high‐yielding rice. Breed Sci. 66, 659–667. 28163581 10.1270/jsbbs.16120 PMC5282764

[pbi14102-bib-0056] Xu, R. , Duan, P. , Yu, H. , Zhou, Z. , Zhang, B. , Wang, R. , Li, J. *et al*. (2018) Control of grain size and weight by the OsMKKK10‐OsMKK4‐OsMAPK6 signaling pathway in rice. Mol. Plant, 11, 860–873. 29702261 10.1016/j.molp.2018.04.004

[pbi14102-bib-0057] Yang, X. , Wang, J. , Xia, X. , Zhang, Z. , He, J. , Nong, B. , Luo, T. *et al*. (2021) *OsTTG1*, a WD40 repeat gene, regulates anthocyanin biosynthesis in rice. Plant J. 107, 198–214. 33884679 10.1111/tpj.15285

[pbi14102-bib-0058] Yang, T. , Gu, H. , Yang, W. , Liu, B. , Liang, S. and Zhao, J. (2023) Artificially selected grain shape gene combinations in Guangdong Simiao varieties of rice (*Oryza sativa* L.). Rice, 16, 3. 36648593 10.1186/s12284-023-00620-9 PMC9845460

[pbi14102-bib-0061] Zhang, J. , Yu, Y. , Feng, Y. , Zhou, Y. , Zhang, F. , Yang, Y. , Lei, M. *et al*. (2017) MiR408 regulates grain yield and photosynthesis via a phytocyanin protein. Plant Physiol. 175, 1175–1185. 28904074 10.1104/pp.17.01169 PMC5664482

[pbi14102-bib-0063] Zhang, L. , Wang, R. , Xing, Y. , Xu, Y. , Xiong, D. , Wang, Y. and Yao, S. (2021a) Separable regulation of *POW1* in grain size and leaf angle development in rice. Plant Biotechnol. J. 19, 2517–2531. 34343399 10.1111/pbi.13677 PMC8633490

[pbi14102-bib-0064] Zhang, D. , Zhang, M. and Liang, J. (2021b) RGB1 regulates grain development and starch accumulation through its effect on *OsYUC11*‐mediated auxin biosynthesis in rice endosperm cells. Front. Plant Sci. 12, 585174. 33868323 10.3389/fpls.2021.585174 PMC8045708

[pbi14102-bib-0065] Zhang, P. , Zhu, C. , Geng, Y. , Wang, Y. , Yang, Y. , Liu, Q. , Guo, W. *et al*. (2021c) Rice and Arabidopsis homologs of yeast CHROMOSOME TRANSMISSION FIDELITY PROTEIN 4 commonly interact with Polycomb complexes but exert divergent regulatory functions. Plant Cell, 33, 1417–1429. 33647940 10.1093/plcell/koab047 PMC8254485

[pbi14102-bib-0067] Zhao, Y. , Hu, Y. , Dai, M. , Huang, L. and Zhou, D.‐X. (2009) The WUSCHEL‐related homeobox gene *WOX11* Is required to activate shoot‐borne crown root development in rice *Plant* . Cell, 21, 736–748. 10.1105/tpc.108.061655 PMC2671696 19258439

[pbi14102-bib-0068] Zhao, Y. , Cheng, S. , Song, Y. , Huang, Y. , Zhou, S. , Liu, X. and Zhou, D.‐X. (2015) The interaction between rice ERF3 and WOX11 promotes crown root development by regulating gene expression involved in cytokinin signaling. Plant Cell, 27, 2469–2483. 26307379 10.1105/tpc.15.00227 PMC4815106

[pbi14102-bib-0069] Zhong, H. , Liu, C. , Kong, W. , Zhang, Y. , Zhao, G. , Sun, T. and Li, Y. (2020a) Effect of multi‐allele combination on rice grain size based on prediction of regression equation model. Mol. Genet. Genomics, 295, 465–474. 31863176 10.1007/s00438-019-01627-y

[pbi14102-bib-0070] Zhong, J. , He, W. , Peng, Z. , Zhang, H. , Li, F. and Yao, J. (2020b) A putative AGO protein, OsAGO17, positively regulates grain size and grain weight through OsmiR397b in rice. Plant Biotechnol. J. 18, 916–928. 31529568 10.1111/pbi.13256 PMC7061870

[pbi14102-bib-0071] Zhou, S. , Jiang, W. , Long, F. , Cheng, S. , Yang, W. , Zhao, Y. and Zhou, D.‐X. (2017) Rice homeodomain protein WOX11 recruits a histone acetyltransferase complex to establish programs of cell proliferation of crown root meristem. Plant Cell, 29, 1088–1104. 28487409 10.1105/tpc.16.00908 PMC5466029

