# Supplementary material for: SLG2 specifically regulates grain width through WOX11 ‐mediated cell expansion control in rice
Source: Plant Biotechnol J. 2023 Jun 21;21(9):1904–18. doi: 10.1111/pbi.14102 (PMC10440987; doi:10.1111/pbi.14102)
Supplement: Supplementary file 1 — Figure S1 Observation of brown rice transparency (a) and starch packaging (b) from the mature grains of WT, slg2, GW8‐KOWT and GW8‐KO slg2 . Figure S2 slg2 shows normal grain filling. Figure S3 Expression analysis of cell division and cell expansion genes (a) and grain width‐related genes (b) in slg2 and WT. Figure S4 Comparison of agronomic traits between slg2 and WT. Figure S5 The reduced grain width of slg2 is caused by decreased cell expansion. Figure S6 Knockout of SLG2 results in plants with the slg2 mutant phenotype. Figure S7 Overexpression of SLG2 fully rescues the slg2 mutant phenotype. Figure S8 Protein sequence comparison between SLG2 and HSLG2. Figure S9 The SLG2 homologue HSLG2 shows no regulatory roles in rice development. Figure S10 Verification of the WOX11 knockout lines (WOX11‐KO) by PCR‐based sequencing. Figure S11 The WOX11‐KO lines show reduced grain width similar to the slg2 mutant. Figure S12 Verification of the GW8 knockout lines in the background of WT (GW8‐KOWT) and slg2 (GW8‐KO slg2 ) by PCR‐based sequencing. Figure S13 Statistical analysis of plant height (a), panicle number (b), grain number per main panicle (c), 1000‐grain weight (d), seed setting rate (e), and grain yield per plant (f) of WT, slg2 and GW8‐KO. Figure S14 Expression analysis of cell division and cell expansion genes in WT, slg2, GW8‐KOWT and GW8‐KO slg2 . [file PBI-21-1904-s001.docx]

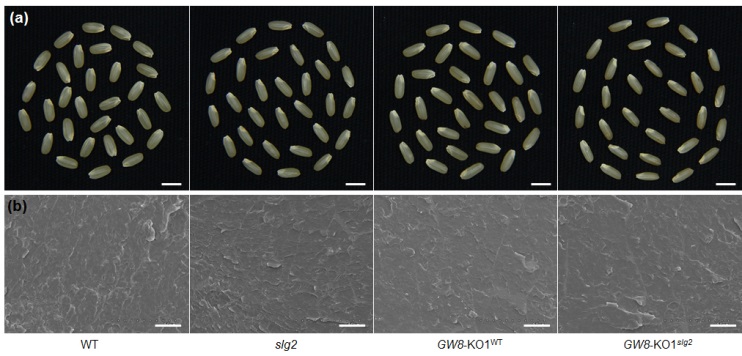


**Figure S1.** Observation of brown rice transparency **(a)** and starch packaging **(b)** from mature grains of WT, *slg2*, *GW8*-KO^WT^ and *GW8*-KO*^slg2^*. Bars = 5 mm **(a)** and 20 μm **(b)**.


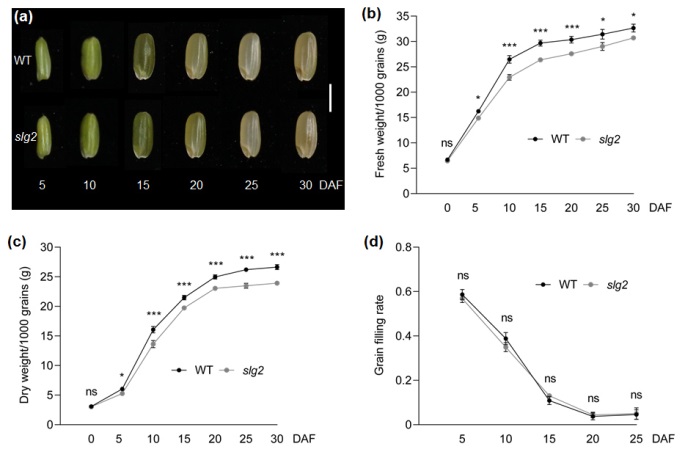


**Figure S2.** *slg2* shows normal grain filling.

**(a)** Comparison of caryopsis development between *slg2* and WT. Bar = 5 mm.

**(b-c)** Comparison of fresh weight **(b)** and dry weight **(c)** between *slg2* and WT at different stages of caryopsis development. Developing ovaries were collected at the indicated days. Data are means ± SD (*n* = 3). *: P < 0.05, ***: P < 0.001, ns: no significant difference (Student’s *t* test).

**(d)** Comparison of grain filling rate between *slg2* and WT. Data are means ± SD (*n* = 3). ns: no significant difference (Student’s *t* test).


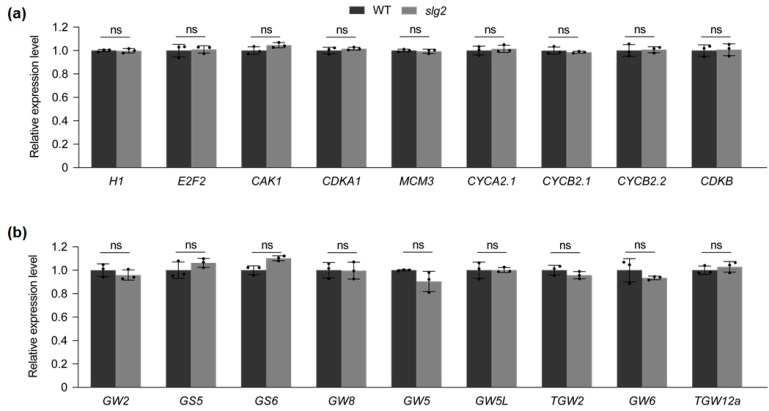


**Figure S3.** Expression analysis of cell division and cell expansion genes **(a)** and grain width-related genes **(b)** in *slg2* and WT. RNA isolated from young panicles of 2-3 mm in length is used for RT-qPCR. *OsActin* is used as the internal control. The transcript levels are normalized against WT, which is set to 1. Data are means ± SD (*n* = 3). ns: no significant difference (Student’s *t* test).


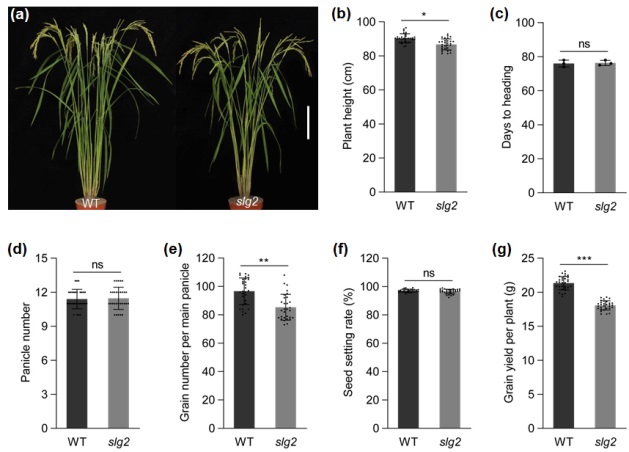


**Figure S4.** Comparison of agronomic traits between *slg2* and WT.

**(a)** Plant morphology of *slg2* and WT at the grain filling stage. Bar = 15 cm.

**(b-g)** Statistical analysis of plant height **(b)**, days to heading **(c)**, panicle number **(d)**, grain number per main panicle **(e)**, seed setting rate **(f)**, and grain yield per plant **(g)**. Data are means ± SD (*n* = 30). *: *P* < 0.05, **: *P* < 0.01, ***: *P* < 0.001, ns: no significant difference (Student’s *t* test).


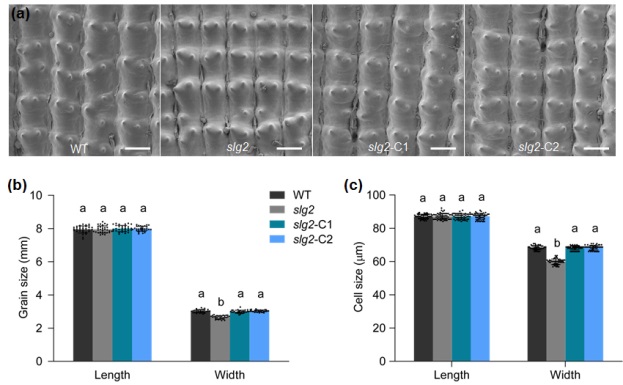


**Figure S5.** The reduced grain width of *slg2* is caused by decreased cell expansion.

**(a)** SEM images of outer glume surfaces of WT, *slg2* and two complementation lines (*slg2*-C). Bars = 75 μm.

**(b)** Statistical analysis of grain size of WT, *slg2* and *slg2*-C. Data are means ± SD (*n* = 30). Bars followed by different letters represent significant difference at 5%.

**(c)** Statistical analysis of cell size in the spikelet hulls of WT, *slg2* and *slg2*-C. The spikelet hulls are sampled before anthesis. Data are means ± SD (*n* = 50). Bars followed by different letters represent significant difference at 5%.


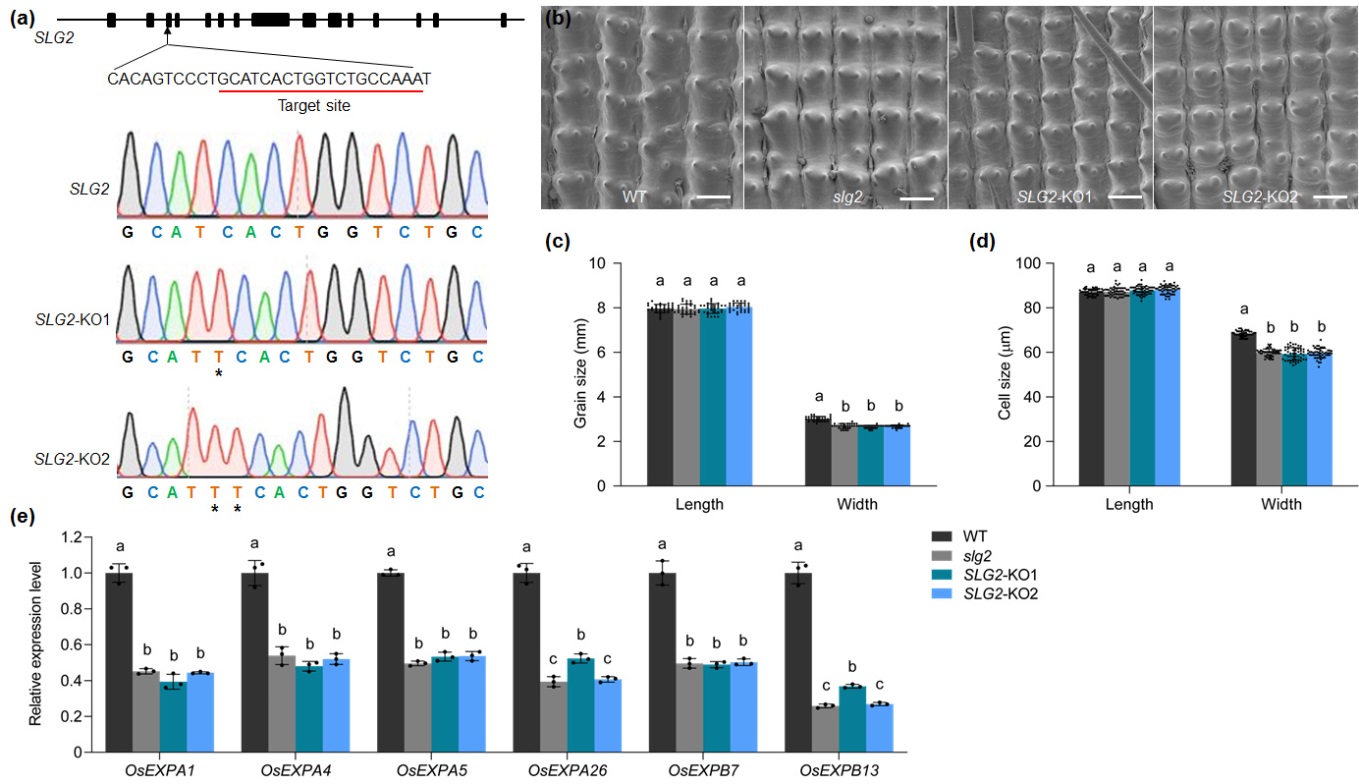


**Figure S6.** Knockout of *SLG2* results in plants with the *slg2* mutant phenotype.

**(a)** Creation of *SLG2* knockout transgenic line by the CRISPR-Cas9 genome editing system. The target site is red-underlined. Two representative transgenic lines (abbreviated as *SLG2*-KO1 and *SLG2*-KO2, respectively) are generated from ZY66 genetic background. The asterisks indicate inserted nucleotides.

**(b)** SEM images of outer glume surfaces of WT, *slg2* and *SLG2-*KO. Bars = 75 μm.

**(c)** Statistical analysis of grain size of WT, *slg2* and *SLG2-*KO. Data are means ± SD (*n* = 30). Bars followed by different letters represent significant difference at 5%.

**(d)** Statistical analysis of cell size in the spikelet hulls of WT, *slg2* and *SLG2-*KO. The spikelet hulls are sampled before anthesis. Data are means ± SD *(n* = 50). Bars followed by different letters represent significant difference at 5%.

**(e)** Expression analysis of cell expansion genes in WT, *slg2* and *SLG2*-KO. RNA isolated from young panicles of 2-3 mm in length is used for RT-qPCR. *OsActin* is used as the internal control. The transcript levels are normalized against WT, which is set to 1. Data are means ± SD (*n* = 3). Bars followed by different letters represent significant difference at 5%.


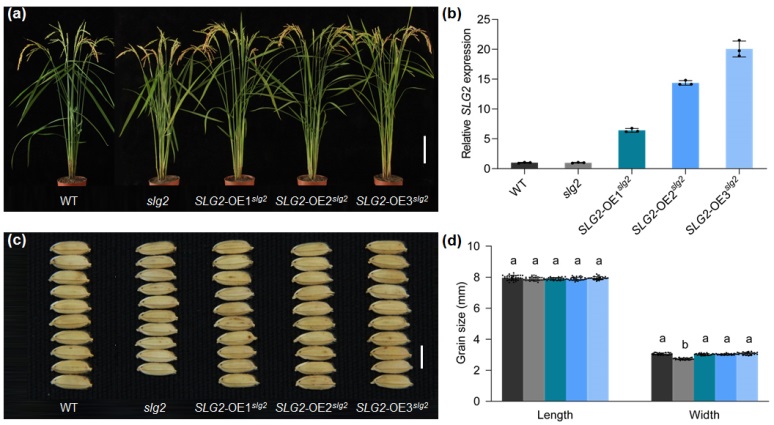


**Figure S7.** Overexpression of *SLG2* fully rescues the *slg2* mutant phenotype.

**(a)** Plant morphology of WT, *slg2* and the *SLG2*-overexpressing lines in the *slg2* background (*SLG2*-OE*^slg2^*). Bar = 15 cm.

**(b)** Expression analysis of *SLG2* in the *SLG2*-OE*^slg2^* lines shown in **(a)**. RNA isolated from young panicles of 2-3 mm in length is used for RT-qPCR. *OsActin* is used as the internal control. The transcripts levels are normalized against *slg2*, which is set to 1. Data are means ± SD (*n* = 3).

**(c)** Grain size comparison among WT, *slg2* and *SLG2*-OE*^slg2^*. Bar = 5 mm.

**(d)** Statistic analysis of grain size of WT, *slg2* and *SLG2*-OE*^slg2^*. Data are means ± SD (*n* = 30). Bars followed by different letters represent significant difference at 5%.


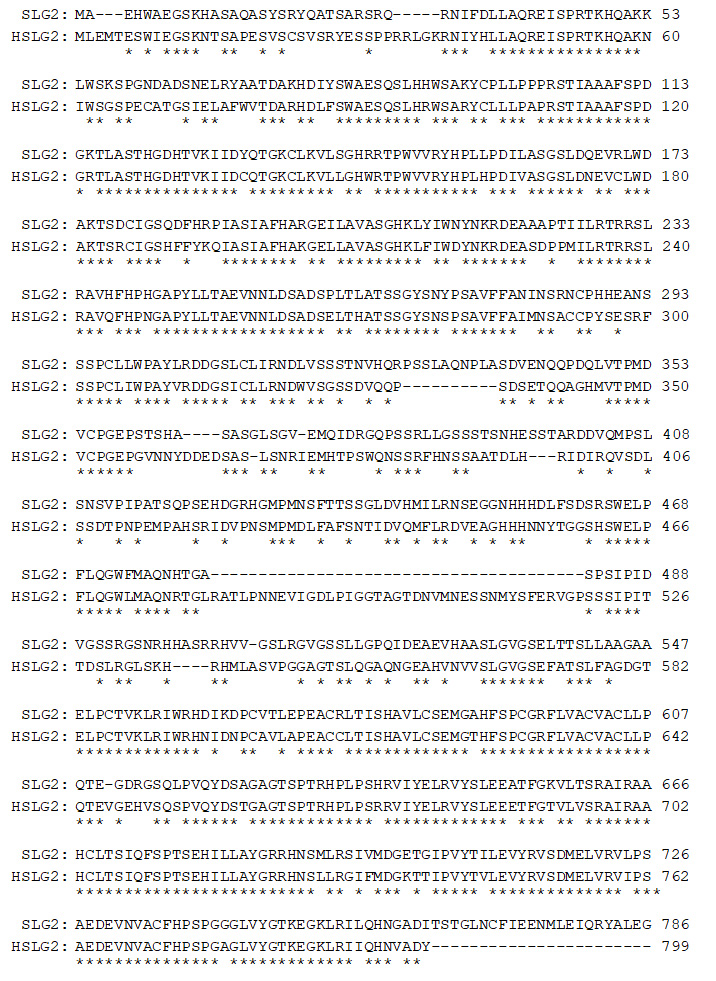


**Figure S8.** Protein sequence comparison between SLG2 and HSLG2. Amino acid alignment is performed by the online Clustal Omega program (https://www.ebi.ac.uk/Tools/msa/clustalo/) with manual curation. The asterisks indicate the same amino acid.


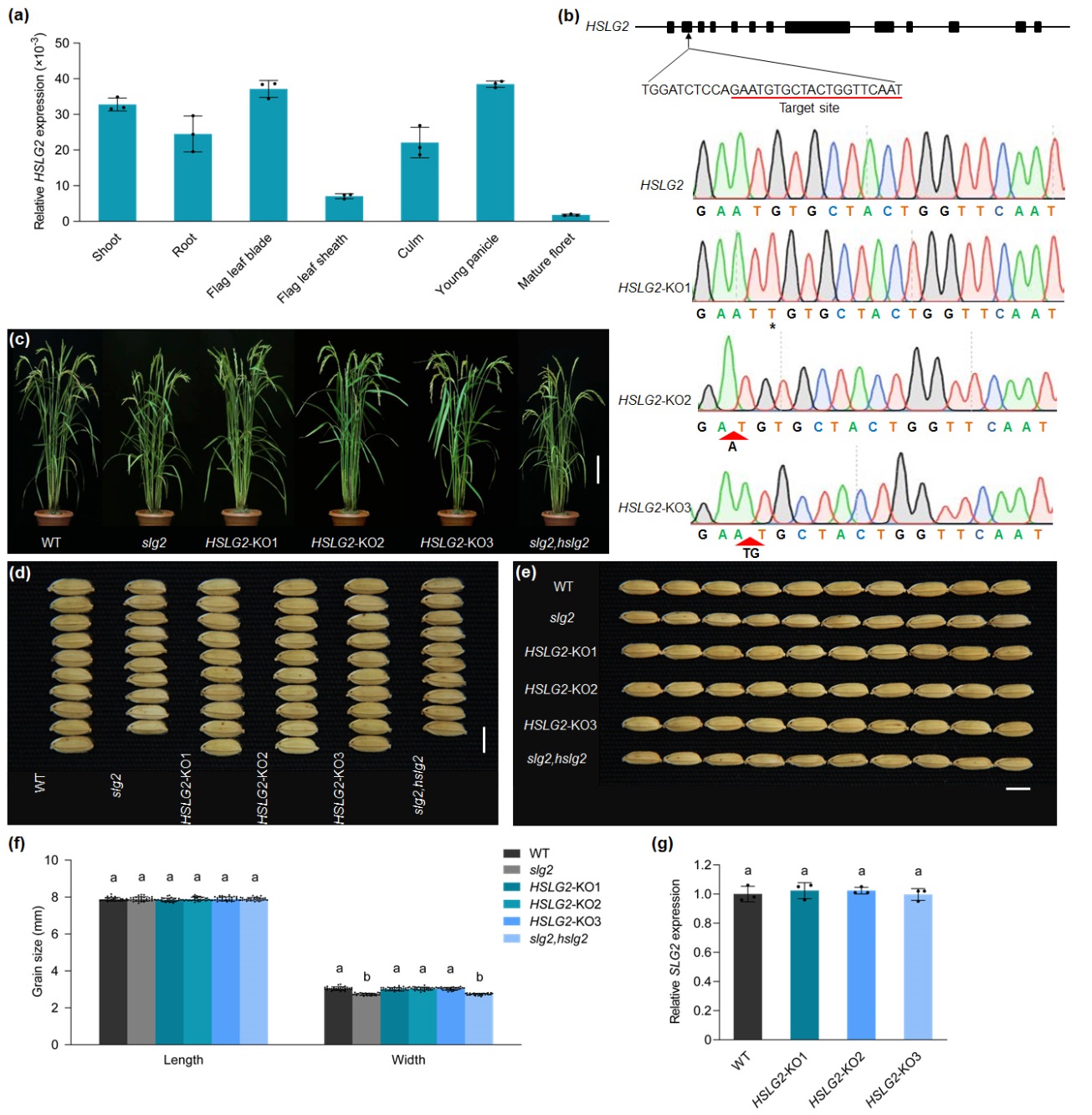


**Figure S9.** The *SLG2* homolog *HSLG2* shows no regulatory roles in rice development.

**(a)** Expression analysis of *HSLG2* in various rice tissues. For RT-qPCR, RNA is isolated from shoots and roots of 7-day-old seedlings, and flag leaf blade, flag leaf sheath, culm, young panicle (2-3 mm in length) and mature floret before anthesis. *OsActin* is used as the internal control. Data are means ± SD (n = 3).

**(b)** Creation of *HSLG2* knockout transgenic line by the CRISPR-Cas9 genome editing system. The target site is red-underlined. The representative transgenic line (abbreviated as *HSLG2*-KO) is generated from ZY66 genetic background. The asterisk indicates inserted nucleotide. The red triangles indicate the positions of deleted nucleotides.

**(c)** Plant morphology of WT, *slg2*, *HSLG2*-KO and the *slg2,hslg2* double mutant plants. Bar = 15 cm.

**(d-e)** Comparison of grain length (d) and grain width (e) between WT, *slg2*, *HSLG2*-KO and the *slg2,hslg2* plants. Bars = 5 mm.

**(f)** Statistical analysis of grain size of WT, *slg2*, *HSLG2*-KO and the *slg2,hslg2* plants. Data are means ± SD (n = 30). Bars followed by different letters represent significant difference at 5%.

**(g)** The transcript levels of *SLG2* in WT and *HSLG2*-KO lines. RNA isolated from young panicles of 2-3 mm in length is used for RT-qPCR. *OsActin* is used as the internal control. The transcript levels are normalized against WT, which is set to 1. Data are means ± SD (n = 3). Bars followed by different letters represent significant difference at 5%.


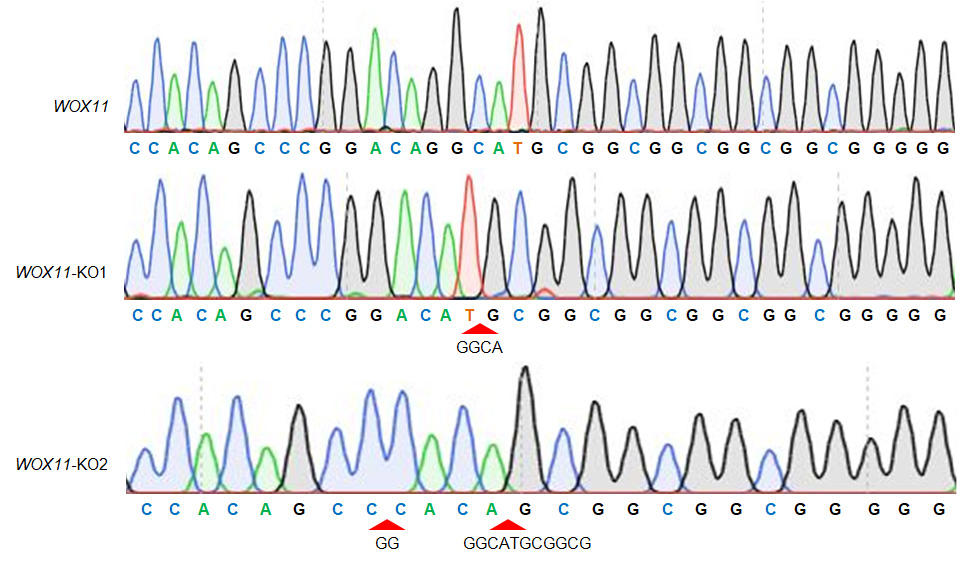


**Figure S10.** Verification of the WOX11 knockout lines (*WOX11*-KO) by PCR-based sequencing. The red triangles indicate the positions of deleted nucleotides.


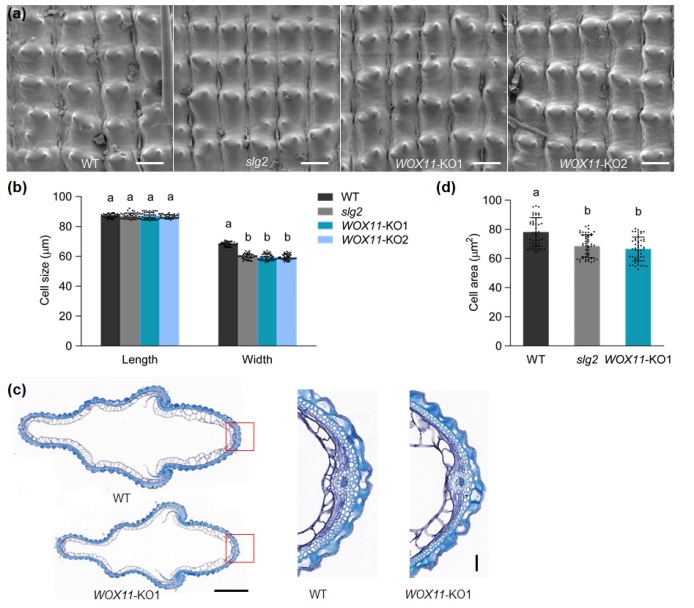


**Figure S11.** The *WOX11-*KO lines show reduced grain width similar to the *slg2* mutant.

**(a)** SEM images of outer glume surfaces of WT, *slg2* and *WOX11-*KO. Bars = 75 μm.

**(b)** Statistical analysis of cell size of WT, *slg2* and *WOX11*-KO. Data are means ± SD (*n* = 50). Bars followed by different letters represent significant difference at 5%.

**(c-d)** Statistical analysis of cell area of WT, *slg2* and *WOX11*-KO1. Cross sections are made from the central parts of the spikelet hulls (c), and cell area is determined by the Image J software. Data are means ± SD (*n* = 50). Bars followed by different letters represent significant difference at 5%. In **(c)**, bars = 500 μm for the left images and 50 μm for the right images**,** and the boxes indicate the positions of the enlarged images on the right.


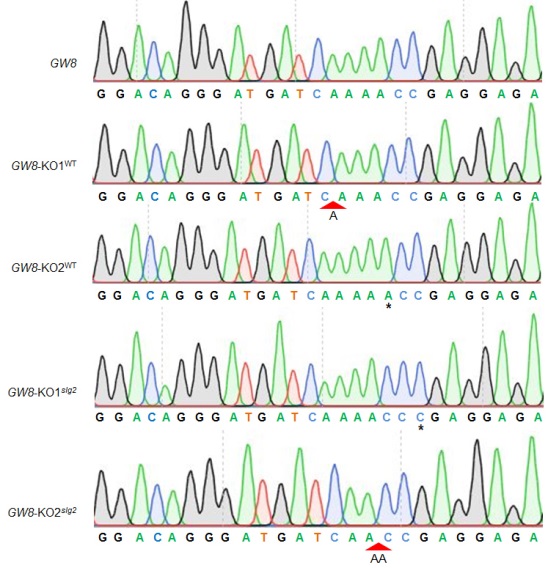


**Figure S12.** Verification of the *GW8* knockout lines in the background of WT (*GW8*-KO^WT^) and *slg2* (*GW8*-KO*^slg2^*) by PCR-based sequencing. The red triangles indicate the positions of deleted nucleotides, and the asterisks indicate inserted nucleotide.


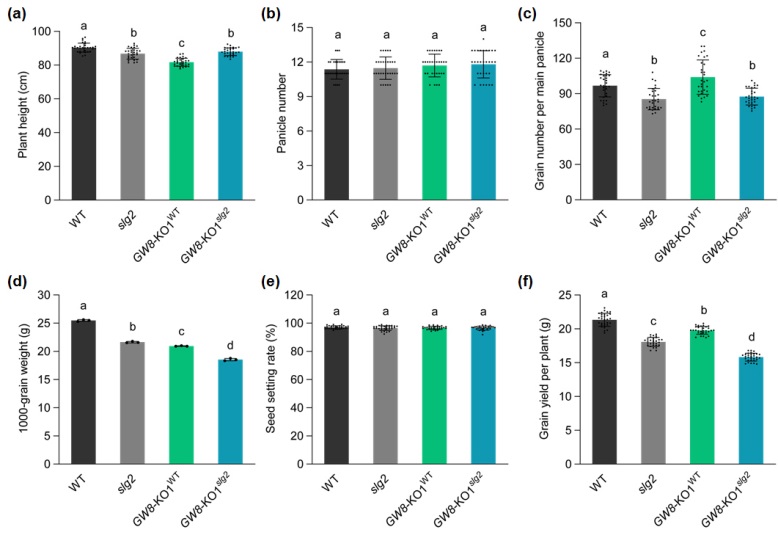


**Figure S13.** Statistical analysis of plant height **(a)**, panicle number **(b)**, grain number per main panicle **(c)**, 1000-grain weight **(d)**, seed setting rate **(e)**, and grain yield per plant **(f)** of WT, *slg2* and *GW8*-KO. Data are means ± SD (*n* = 30). Bars followed by different letters represent significant difference at 5%.


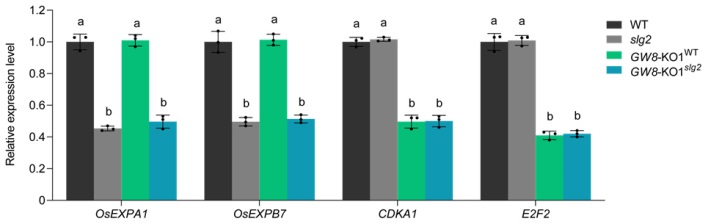


**Figure S14.** Expression analysis of cell division and cell expansion genes in WT, *slg2*, *GW8*-KO^WT^ and *GW8*-KO*^slg2^*. RNA isolated from young panicles of 2-3 mm in length is used for RT-qPCR. *OsActin* is used as the internal control. The transcript levels are normalized against WT, which is set to 1. Data are means ± SD (*n* = 3). Bars followed by different letters represent significant difference at 5%.
